# Gut Microbial Succession Patterns and Metabolic Profiling during Pregnancy and Lactation in a Goat Model

**DOI:** 10.1128/spectrum.02955-22

**Published:** 2023-01-26

**Authors:** Ke Zhang, Gongwei Liu, Yujiang Wu, Ting Zhang, Mengmeng Guo, Yu Lei, Xi Cao, Langda Suo, Daniel Brugger, Xiaolong Wang, Yuxin Yang, Yulin Chen

**Affiliations:** a Key Laboratory of Animal Genetics, Breeding and Reproduction of Shaanxi Province, College of Animal Science and Technology, Northwest A&F University, Yangling, China; b Institute of Animal Sciences, Tibet Academy of Agricultural and Animal Husbandry Sciences, Lhasa, China; c Key Laboratory of Animal Genetics and Breeding on Tibetan Plateau, Ministry of Agriculture and Rural Affairs, Lhasa, China; d Key Laboratory of Animal Biotechnology, Ministry of Agriculture, College of Veterinary Medicine, Northwest A&F University, Yangling, China; e Institute of Animal Nutrition and Dietetics, Vetsuisse-Faculty, University of Zurich, Zurich, Switzerland; Chengdu University

**Keywords:** estradiol, hindgut microbiome, metabolome, progesterone, reproductive cycle, ruminants

## Abstract

The maternal gut microbiome affects the duration of pregnancy, delivery, and lactation. It also coordinates the stability of maternal metabolism by regulating and modulating inflammatory cytokines and reproductive hormones. This has been shown in several species; however, the situation in ruminants remains a black box. Here, we aimed to elucidate the relationship between the hindgut microbiota, metabolism, and reproductive hormones in domestic goats (Capra hircus) during nonpregnancy, pregnancy, and lactation stages. The hindgut microbiota was altered during these three stages, with a drastic decrease in the abundance of *Family_XIII_AD3011_group* in the second and third trimesters of pregnancy. Additionally, a decline in the abundance of *Christensenellaceae_R-7_group* and *Turicibacter* was observed from the nonpregnancy stage to late gestation. *Family_XIII_AD3011_group* and *Paeniclostridium* were strongly correlated with decreased fecal estradiol and progesterone. Furthermore, we generated a metabolome atlas of the gut and serum from nonpregnancy to lactation to reveal the specific metabolic fingerprints of each physiological stage. Several specific gut metabolites, including carnitine C8:1, γ-aminobutyric acid, and indole-3-carboxylic acid, were negatively correlated with the fecal and serum estradiol concentrations. In contrast, 2′-deoxyinosine, deoxyadenosine, and 5′-deoxyadenosine were positively correlated with the fecal and serum estradiol concentrations. The levels of 2′-deoxyinosine, deoxyadenosine, and 5′-deoxyadenosine in fecal samples were positively correlated with *Family_XIII_AD3011_group*. Other serum metabolites, such as (±)12-HEPE (hydroxy eicosapentaenoic acid), (±)15-HEPE, (±)18-HEPE, cytidine, uracil, and 5-hydroxyindole-3-acetic acid, were negatively correlated with the serum concentrations of estradiol and progesterone. Finally, *Corynebacterium* and *Clostridium_sensu_stricto_1* in the fecal samples were positively correlated with the abundance of 11,12-EET (epoxy-eicosatrienoic acid), (±)18-HEPE, (±)15-HEPE, and (±)12-HEPE in the serum.

**IMPORTANCE** Our findings revealed that the activity of *Family_XIII_AD3011_group* and *Corynebacterium* is strongly correlated with the beneficial regulation of physiological hormones and metabolic changes during pregnancy and lactation. These findings are key for guiding targeted microbial therapeutic approaches to modulate microbiomes in gestating and lactating mammals.

## INTRODUCTION

Pathological changes in hormone secretion and immune responses during the second trimester of pregnancy (Pre) are commonly known as the maternal metabolic disorder syndrome (1–3), which is characterized by insulin resistance (4), inflammation (5), and oxidative stress (6). The syndrome is common in several mammalian species, including pigs (7), humans (8), sheep (9), and mice (10, 11), where the pregnancy is accompanied by an increase in mucosa-degrading microbiota (12, 13). The associated disturbance of gut microbial populations further promotes the increase of gut permeability (14). An increase in gut permeability in pregnant women was shown to be positively correlated with a general decrease in the gut microbiota (15), which is one of the potential mechanisms by which gut microbiota alters the maternal metabolome. Understanding the interactions of the maternal gut microbiota and metabolites during pregnancy and lactation (Lac) in mammals is crucial to develop targeted strategies to address the maternal metabolic disorder syndrome.

The gut microbiome affects the stability of maternal metabolism via short-chain fatty acids (SCFAs), inflammatory cytokines, bile acid metabolism, and metabolic hormones (16). The bile acids and SCFAs that are arising from the maternal microbial metabolome can penetrate the placental barrier post absorption, thereby promoting the development and growth of the fetal organs (17). 5-Hydroxytryptamine (5-HT) is an important neurotransmitter found in the brain and intestines of mammals (18). Diet affects the secretion of 5-HT in the intestine, which regulates intestinal movement, mood, appetite, sleep, and cognitive functions (19). A general antibiotic-induced depletion of mice’s microbiota impaired serotonin biosynthesis and led to decreased intestinal motility (20). Therefore, gut microbiota could affect the host’s behavior via its serotonin metabolism.

In humans and mice, the prenatal period is characterized by an increase in serum hormones like progesterone (Prog) and proinflammatory factors, as well as decreased immune activity, which alter gut function and microbiota composition (21–23). Specifically, estradiol (E2) and Prog alter the composition of the gut microbiome by regulating the microbial metabolism and growth of pathogenic microbes (24). The drastic changes in ovarian hormones before and after delivery further alter gut contractility and transport through mucosal membranes, which improves the absorptive capacity for dietary nutrients and energy. This increases the risk of obesity during pregnancy without proper dietary management (25). In addition, elevated E2 and Prog levels during pregnancy change intestinal function and microbiome composition, leading to an increase in the susceptibility to pathogens (26, 27). The concept of a functional interaction between gut microbes and reproductive hormones has been well established in pigs (7, 28) and humans (21, 29). In marked contrast, despite the intensive microbiome research in ruminants, this group of mammals is still a “black box” with respect to the relationship between gut microbiota and the maternal organism during gestation and lactation.

The present study aimed to investigate the functional interaction between the gut microbial composition, microbial metabolites, and the host’s responses during pregnancy and lactation of domestic goats (*Capra hircus*). Specifically, we characterized the changes in the hindgut microbiota and associated metabolites during nonpregnancy, pregnancy (Pre D0_150), and lactation (Lac D0_56). These findings will help in optimizing the health management of goats and other ruminants during pregnancy and lactation.

## RESULTS

### Pregnancy and lactation progression is precisely ordered by serum hormones and physiological indicators.

**FIG 1:**
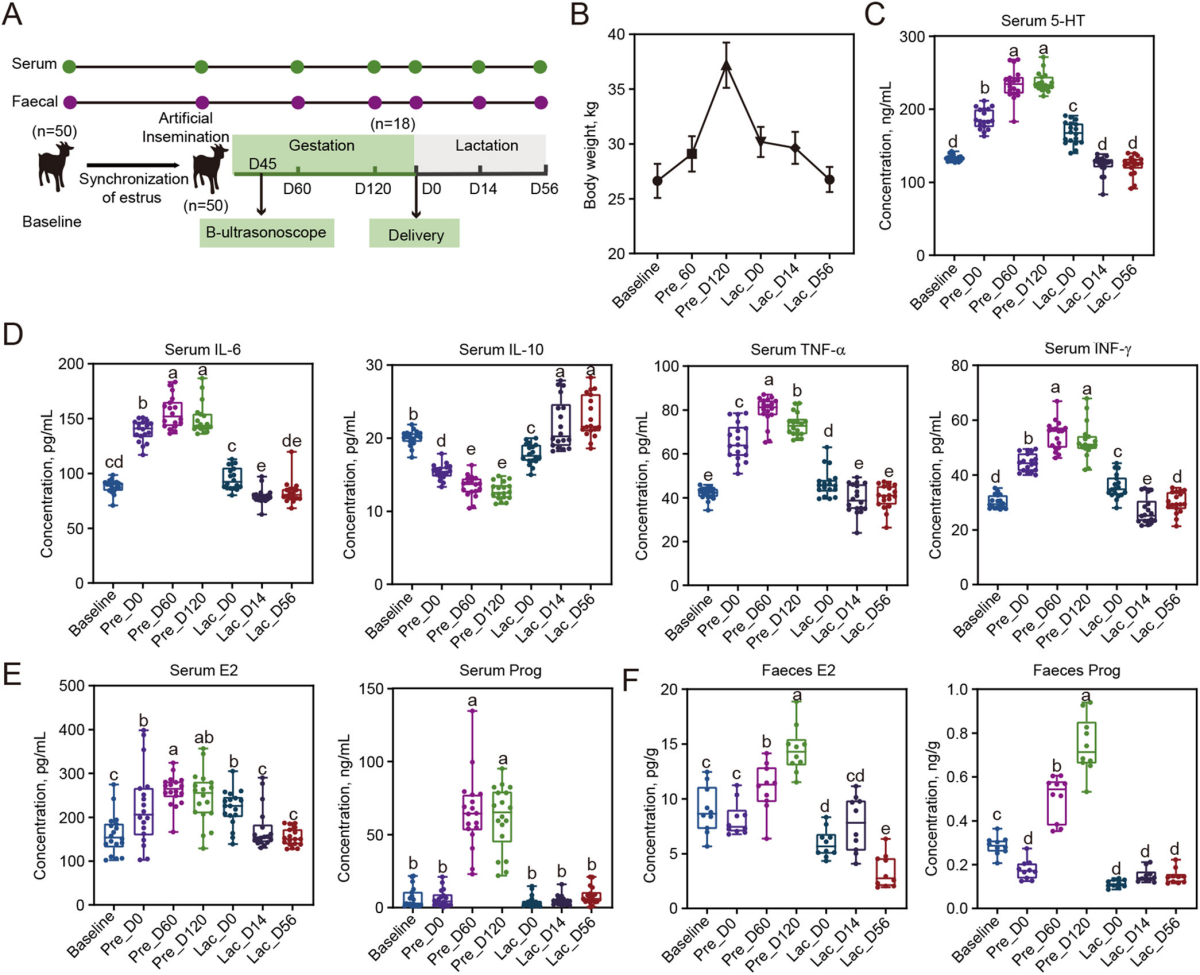
Bodyweight, serum, and fecal parameters during pregnancy, and lactation in goats. (A) The design paradigm; *n *= 18 individuals/group. (B) Body weights; *n *= 18 individuals/group. (C) Serum 5-HT concentration; *n *= 18 individuals/group. (D) Serum inflammatory factors (IL-6, IL-10, TNF-α, and IFN-γ) concentration; *n *= 18 individuals/group. (E) Serum E2 and Prog concentrations; *n *= 18 individuals/group. (F) Fecal E2 and Prog concentrations. *n *= 10 individuals/group. Data differences in goat subjects were assessed using the one-way ANOVA with Tukey’s test. Values followed by the same letter in the same column are not statistically different at *P > *0.05.

The physiological indicators, immunoglobulins, and inflammatory cytokine concentrations were evaluated at various times throughout normal pregnancy and lactation in goats to ascertain how the metabolic properties changed. Between serum collection at 0 (Pre_D0) and 120 days (Pre_D120), the concentrations of 5-HT, interleukin-6 (IL-6), tumor necrosis factor-α (TNF-α), interferon (IFN-γ), and body weight were considerably high, but between Pre_D120 and day 14 of lactation (Lac_D14), these parameters were dramatically dropped (*P < *0.001; Fig. 1B to C). Contrarily, IL-10 was dramatically reduced from Pre_D0 to Pre_D120 and then significantly increased from Pre_D120 to Lac_D14 (*P < *0.001; Fig. 1D). From Lac_D14 to Lac_D56, these indices showed no discernible alterations (*P < *0.001; Fig. 1B to D). Furthermore, the concentrations of total cholesterol (TC), high-density lipoprotein (HDL), complement 3 (C3), complement 4 (C4), immunoglobulin A (IgA), immunoglobulin G (IgG), and immunoglobulin M (IgM) in serum were significantly decreased at gestation (Pre_D60 and Pre_D120) compared to the Baseline (*P < *0.01; Fig. S1 in the supplemental material). Triacylglycerol (TG) and low-density lipoprotein (LDL) were significantly decreased during lactation (Lac_D0, Lac_D14 and Lac_D56) compared to the Baseline (*P < *0.01; Fig. S1). In addition, the assessment of the concentration of hormones in the serum and fecal samples revealed that serum E2 was significantly increased at Pre_D0 and Lac_D0 compared to Baseline (*P < *0.01; Fig. 1E), while feces E2 was significantly increased at gestation (Pre_D60 and Pre_D120) compared to Baseline (*P < *0.001; Fig. 1F). Specifically, the serum and fecal Prog concentrations were significantly increased at gestation (Pre_D60 and Pre_D120) compared to Baseline (*P < *0.001; Fig. 1E to F). Overall, these findings indicate that the goats had a lower level of immunity and a higher level of inflammation during pregnancy.

### The dynamics of goat gut microbiota during pregnancy and lactation progression.

**FIG 2:**
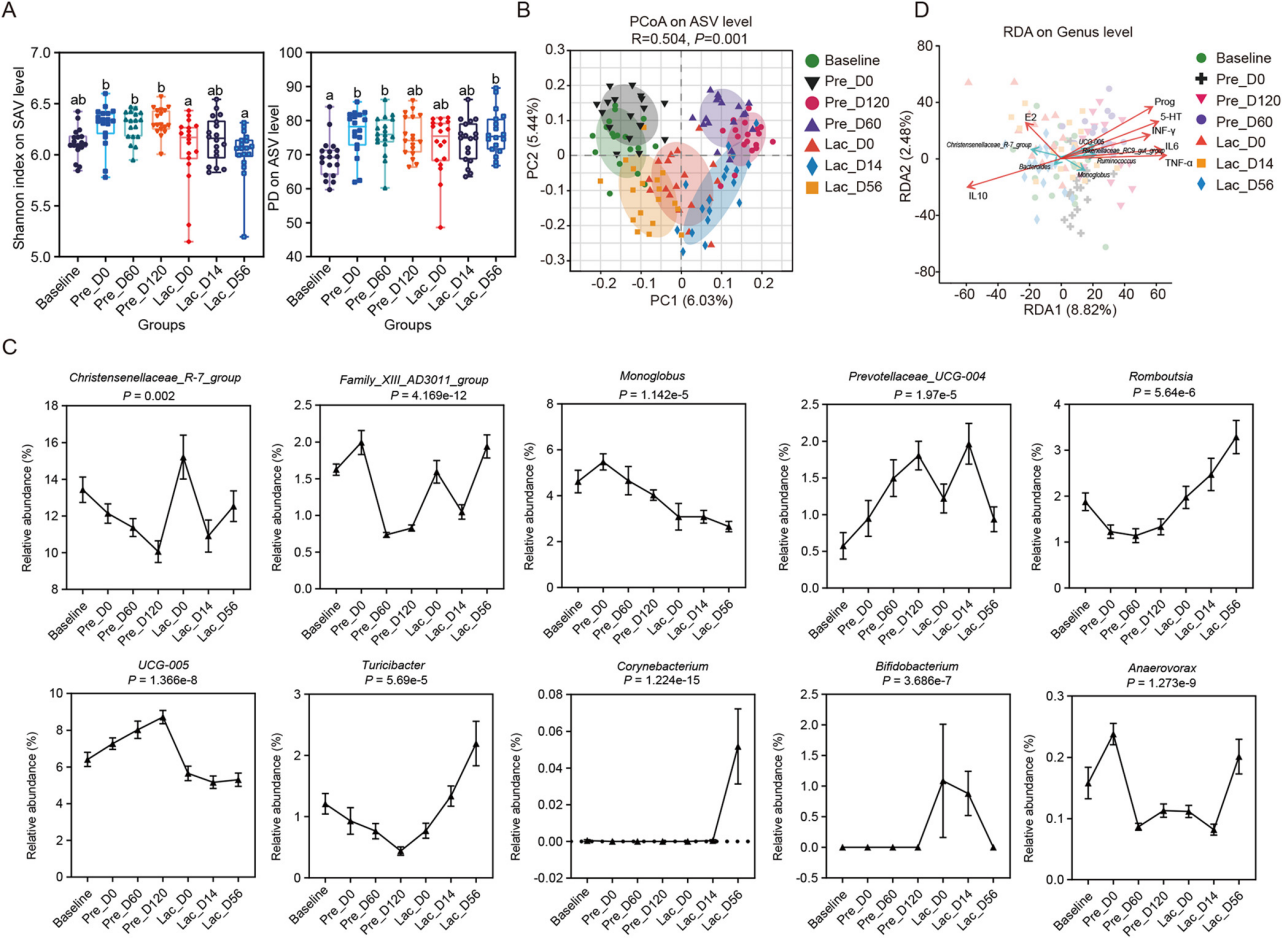
Gut microbiota composition during pregnancy and lactation in goats. (A) Comparison of alpha diversity indices of bacterial communities at the ASV level. (B) Principle coordinate analysis (PCoA) plot of gut microbiota based on the ASV matrix; β-diversity by ANOSIM analysis. (C) The changes in gut microbiota composition during the different stages of pregnancy and lactation. The nonparametric Kruskal-Wallis tests followed by post hoc Tukey-Kramer with Benjamin-Holmes with FDR correction. (D) Correlation between microbiota composition, serum inflammatory factors, and reproductive hormone by redundancy analysis at different stages of pregnancy and lactation. The relationship between element points is represented by distance—the closer the distance, the similarity in sample composition. The obtuse angle represents negative correlation, while the acute angle represents positive correlation. *n *= 18 individuals/group.

A total of 126 fecal samples were collected from 18 goats during pregnancy and lactation to determine the dynamics of their gut microbiota. A total of 4,083,647 amplicon sequence reads (average 32,409 reads per sample) were obtained from the sequencing of the V3 and V4 regions of the 16S rRNA genes of the gut microbiota present in the fecal samples. The amplicon sequence variants (ASVs) clustered at 97% pairwise sequence identity, and taxonomies revealed the richness and diversity of gut microbiota reflected in the rarefaction curves (Fig. S2A) and Chao index (Fig. S2B). The Shannon and PD indexes of ASV were only significantly increased during gestation compared to Baseline (Fig. 2A). Principal-coordinate analysis (PCoA) and analysis of similarities (ANOSIM) analysis based on the Bray-Curtis distances revealed that the gut microbiota was grouped between seven different time points (*R* = 0.504, *P = *0.001; Fig. 2B). The different phylogenetic levels, including the phylum and family of the gut microbiota 16S rRNA-gene amplicon analysis, are presented in Fig. S2C to E. At the phylum level, the relative abundances of *Spirochaetota* and *Fibrobacterota* from Baseline to Lac_D14 were increased (corrected *P = *0.005; Fig. S2C). In addition, the relative abundances of *Patescibacteria* and *Elusimicrobiota* were significantly increased (corrected *P < *0.001) and decreased (corrected *P = *0.02) from Baseline to Pre_D60, respectively. Besides, the relative abundances of *Actinobacteriota* were increased from Pre_D60 to Lac_D0 (corrected *P < *0.001; Fig. S2C). At the family level, *Rikenellaceae* was the most abundant, while *Anaerovoracaceae* and *Bacteroidaceae* were the least abundant at Pre_D60 (corrected *P < *0.001; Fig. S2D). *Oscillospiraceae* was the most abundant, while *Anaerovoracaceae* was the least abundant at Pre_D120 (corrected *P < *0.001; Fig. S2D). Furthermore, the mean relative abundances of *Christensenellaceae* and *Lachnospiraceae* were the highest at Lac_D0 (corrected *P < *0.001; Fig. S2D). At the same time, the abundance of *Peptostreptococcaceae* and *Erysipelotrichaceae* was significantly increased from Pre_D120 to Lac_D56 (corrected *P < *0.001; Fig. S2D). At the genus level, the abundance of *Prevotellaceae UCG-004* and *UCG-005* bacteria was steadily elevated from the Baseline to the Pre D_120 period (corrected *P < *0.001; Fig. 2C), whereas the abundance of *Christensenellaceae R-7* and *Turicibacter* bacteria declined (corrected *P < *0.002; Fig. 2C). From Pre_D120 to Lac_D56, *Romboutsia* and *Turicibacter* bacteria became more prevalent (corrected *P < *0.001; Fig. 2C). It is interesting to note that the bacterial abundance of *Family XIII AD3011* reached its lowest value at Pre D_60 and Pre D_120 periods (corrected *P < *0.001; Fig. 2C), *Bifidobacterium* abundance progressively showed a rise after delivery (corrected *P < *0.001; Fig. 2C), and *Corynebacterium* abundance was essentially undetectable until Lac_D56 (corrected *P < *0.001; Fig. 2C). Overall, the core gut microbiota composition of goats was profoundly altered during pregnancy and lactation.

### Hormone levels have a strong correlation with changes in gut microbiota.

**FIG 3:**
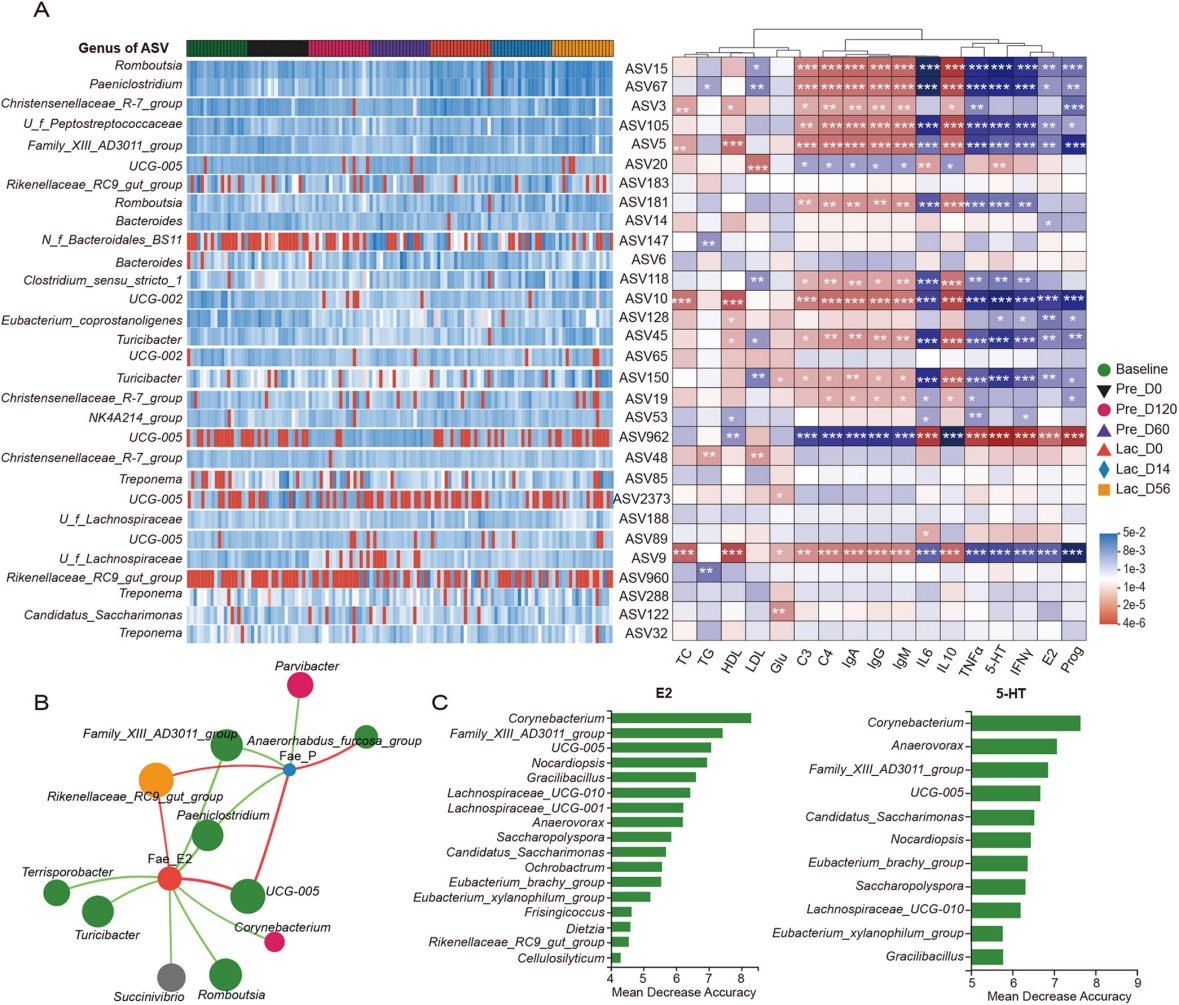
Correlations between the gut microbiota composition at ASV level and the concentration of serum parameters during pregnancy and lactation. (A) Heatmap correlations between microbiota ASV and serum physiological index. Color denotes positive (blue) and negative (red) correlation values. *, *P < *0.05, **, *P ≤ *0.01, and ***, *P ≤ *0.001. (B) Network analysis of correlations between microbiota core genera and feces E2 and Prog concentration. The green line represents negative correlation, and the red line represents positive correlation; *n *= 10 individuals/group. (C) The 18 genera markers were selected as the optimal marker set by random forest models based on E2 and 5-HT concentration.

To identify the relationship between serum hormones and microbial community composition, canonical correlation analysis (CCA) was used. The Prog, 5-HT, IFN-γ, IL-6, and TNF-α were distributed in the second quadrant, E2 in the first, while IL-10 was in the third (Fig. 2D). *UCG-005*, *Rikenellaceae_RC9_gut_group*, *Ruminococcus*, and *Monoglobus* were negatively correlated with E2 and IL-10 (Fig. 2D). *Christensenellaceae_R-7_group* and *Bacteroides* were negatively correlated with 5-HT, Prog, IFN-γ, IL-6, and TNF-α (Fig. 2D). Remarkably, several of the significant correlations were ascribable to microbe colonizers (Fig. 3A). At the family level, *Anaerovoracaceae*, *Peptostreptococcaceae*, and *Erysipelotrichaceae* were strongly correlated with decreased levels of IL-6, TNF-α, HT5, IFN-γ, E2, and Prog in the serum (Fig. S3), while *Anaerovoracaceae*, *Peptostreptococcaceae*, and *Erysipelotrichaceae* were strongly correlated with increased concentrations of C3, C4, IgA, IgG, IgM, and IL-10 in the serum (Fig. S3). At the genus level, *Romboutsia* (ASV 15 and ASV 181), *Paeniclostridium* (ASV 67), *Turicibacter* (ASV 150 and ASV 45), and *UCG-002* (ASV 10) induced the most systemic changes, including an increased concentration of 5-HT, IFN-γ, IL-6, and TNF-α and lowered concentration of C3, C4, IgA, IgG, IgM, and IL-10 in the serum (Fig. 3A).

The Spearman correlation network further revealed a correlation between bacterial colonizers and fecal E2 and Prog (Fig. 3B). Interestingly, *Family_XIII_AD3011_group* and *Paeniclostridium* were strongly correlated with decreased fecal E2 and Prog (*r* < −0.6, *P < *0.05), while *Rikenellaceae_RC9_gut_group*, *UCG-005*, *UCG-010*, and *Butyricicoccaceae* were strongly correlated with an increased fecal E2 and P (*r *> 0.6, *P < *0.05; Fig. 3B). The abundance of *Turicibacter*, *Romboutsia*, *Succinivibrio*, and *Corynebacterium* were strongly correlated with decreased fecal E2 (*r* < −0.6, *P < *0.05; Fig. 3B). Random forest analysis further revealed that *Corynebacterium*, *Family_XIII_AD3011_group*, *UCG-005*, and *Anaerovorax* were the most important gut microbial genera in goats during pregnancy and lactation (importance >7) (Fig. 3C). Overall, the abundance of *Family_XIII_AD3011_group*, *UCG-005*, and *Corynebacterium* were strongly correlated with the systemic changes of serum and fecal hormones.

### Perturbations of gut metabolites during pregnancy and lactation are closely related to changes in reproductive hormones.

**FIG 4:**
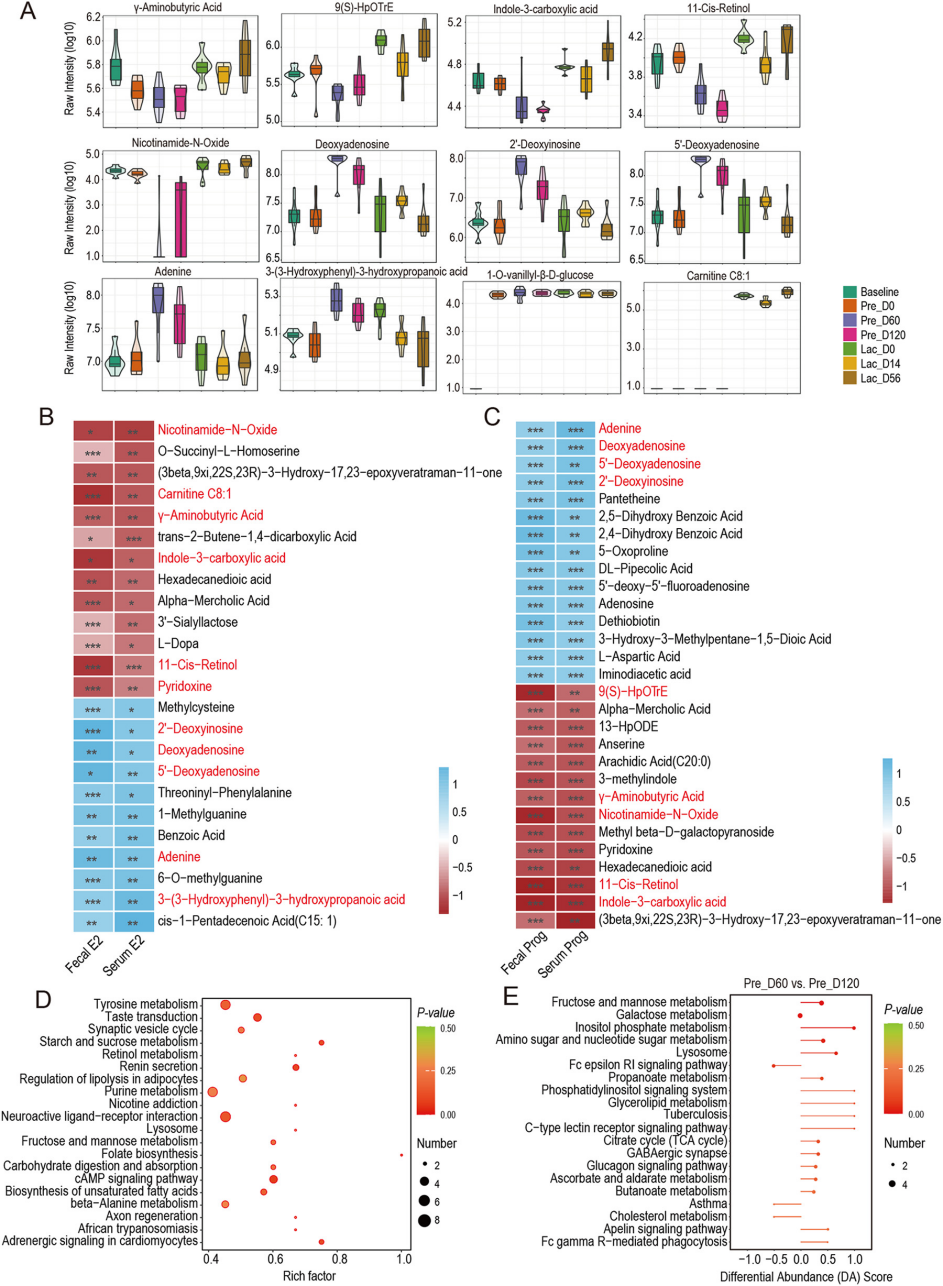
Untargeted metabolites in goat feces during pregnancy and lactation. (A) The relative abundance of significantly different metabolites in feces across the different stages of pregnancy and lactation; *n *= 8 individuals/group. (B) Heatmap correlations between gut metabolites and E2 of feces and serum. Color denotes positive (blue) and negative (red) correlation values. |*r*| ≥ 0.4. *, *P < *0.05, **, *P ≤ *0.01, and ***, *P < *0.001. (C) Heatmap correlations between gut metabolites and Prog of feces and serum. Color denotes positive (blue) and negative (red) correlation values. |*r*| ≥ 0.4. *, *P < *0.05, **, *P ≤ *0.01, and ***, *P ≤ *0.001. (D) Metabolic pathways are altered during pregnancy and lactation. Red dots denote pregnancy-related pathways at *P < *0.05. (E) Differential abundance (DA) score analysis of KEGG pathway at Pre_D60 verus Pre_D120. The DA score reflects the overall changes in metabolites in the metabolic pathways. A score of 1 indicates the metabolites in the pathway the were upregulated, while −1 indicates they were downregulated. The length of the line segment represents the absolute value of the DA score, while the size of the dot at the end of the line segment represents the number of different metabolites in the pathway.

A total of 610 metabolic features were obtained from 56 fecal samples. Among them, 202 features (33.1%) were altered during pregnancy and lactation. Principal-component analysis (PCA) significantly clustered the metabolic features based on their respective physiological stages (Fig. S4A). The heatmap of differentially detected metabolites revealed separate clustering at Pre_D60 and Pre_D120 and Baseline and Lac_D56 (Fig. S4B). The changes in specific metabolites during pregnancy and lactation were assessed to identify the fecal metabolome. The overall changes in metabolites are listed in Fig. S4C. At the gestation stage, γ-aminobutyric acid, 11-cis-retinolm, 9(*S*)-HpOTrE, indole-3-carboxylic acid, and nicotinamide-*N*-oxide were significantly downregulated (*P < *0.001; Fig. 4A), and the abundance of deoxyadenosine, 2′-deoxyinosine, adenine, and 3-(3-hydroxyphenyl)-3-hydroxypropanoic acid was significantly upregulated (*P < *0.001; Fig. 4A). Intriguingly, 1-*O*-vanillyl-β-d-glucose was not detected at Baseline (Fig. 4A), and carnitine C8:1 was only enriched during lactation (Fig. 4A). The spearman correlation network analysis further revealed that carnitine C8:1, γ-aminobutyric acid, nicotinamide-*N*-oxide, pyridoxine, 11-cis-retinol, and indole-3-carboxylic acid were negatively correlated with the E2 concentrations of feces and serum (*r* < −0.4, *P < *0.05; Fig. 4B). The abundance of 2′-deoxyinosine, deoxyadenosine, 5′-deoxyadenosine, adenine, and 3-(3-hydroxyphenyl)-3-hydroxypropanoic acid was positively correlated with the E2 concentrations of feces and serum (*r *> 0.4, *P < *0.05; Fig. 4B). Equally important, the abundance of 9(*S*)-HpOTrE, γ-aminobutyric acid, nicotinamide-*N*-oxide, 11-cis-retinol, and indole-3-carboxylic acid was negatively correlated with the Prog concentrations of feces and serum (*r* < −0.4, *P < *0.05; Fig. 4C). The abundance of adenine, deoxyadenosine, 5′-deoxyadenosine, and 2′-deoxyinosine was positively correlated with the Prog concentrations of feces and serum (*r *> 0.4, *P < *0.05; Fig. 4C). Taken together, these results indicate that key metabolic composition and abundance are correlated with changes of the host hormone level.

The functional groups of metabolites altered during pregnancy and lactation were determined by examining the global pathway changes of the 610 annotated compounds. The 107 mapped KEGG pathways (Table S5) were mainly associated with the following pathways: cAMP signaling pathway (ko04024), renin secretion (ko04924), adrenergic signaling in cardiomyocytes (ko04261), starch and sucrose metabolism (ko00500), folate biosynthesis (ko00790), taste transduction (ko04742), neuroactive ligand-receptor interaction (ko04080), biosynthesis of unsaturated fatty acids (ko01040), and tyrosine metabolism (ko00350) (Fig. 4D). In addition, inositol phosphate metabolism (ko00562), tuberculosis (ko05152), glycerolipid metabolism (ko00561), phosphatidylinositol signaling system (ko04070), and C-type lectin receptor signaling pathway (ko04625) were upregulated in the third trimester of pregnancy (Pre_D120) compared to the first trimester of pregnancy (Pre_D60) (differential abundance [DA] score = 1; Fig. 4E). Taken together, these results found that the changes in key metabolites affect the expression of nutrient metabolism and neural signaling pathways in the host gut.

**FIG 5:**
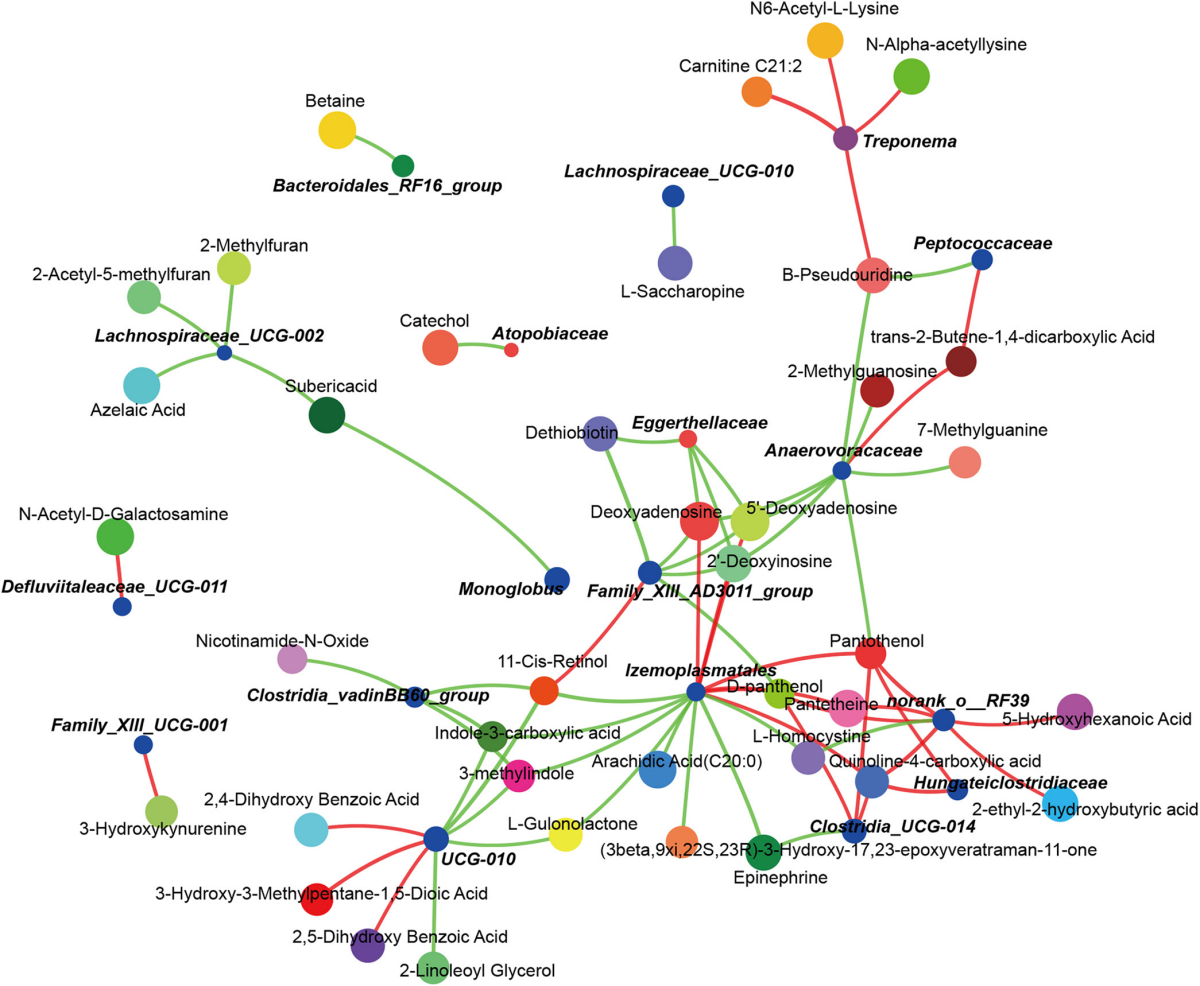
Spearman’s correlation network analysis between the gut microbes and fecal metabolome. The line colors ranged from green (negative correlation) to red (positive correlation) at |*r*| ≥ 0.5 and *P < *0.05 level of significance.

The Spearman correlation network analysis further revealed that the key metabolites, including 2′-deoxyinosine, 5′-deoxyadenosine, deoxyadenosine, dethiobiotin, and d-panthenol, were negatively correlated with *Family_XIII_AD3011_group* (*r* < −0.6, *P < *0.01; Fig. 5) and 11-Cis-retinol was positively correlated with *Family_XIII_AD3011_group* (*r *> 0.6, *P < *0.01; Fig. 5). The metabolites of indole-3-carboxylic acid and 3-methylindole were negatively correlated with *Clostridia_vadinBB60_group* and *UCG-010* (*r* < −0.6, *P < *0.01; Fig. 5). These results indicate that the important microbes of *Family_XIII_AD3011_group* have a relatively great impact on host E2 and Prog levels, and key metabolites related to microbes in the gestation stage may be correlated with physical metabolism and hormone metabolism.

### Association of serum metabolite profiles with reproductive hormones during pregnancy and lactation.

**FIG 6:**
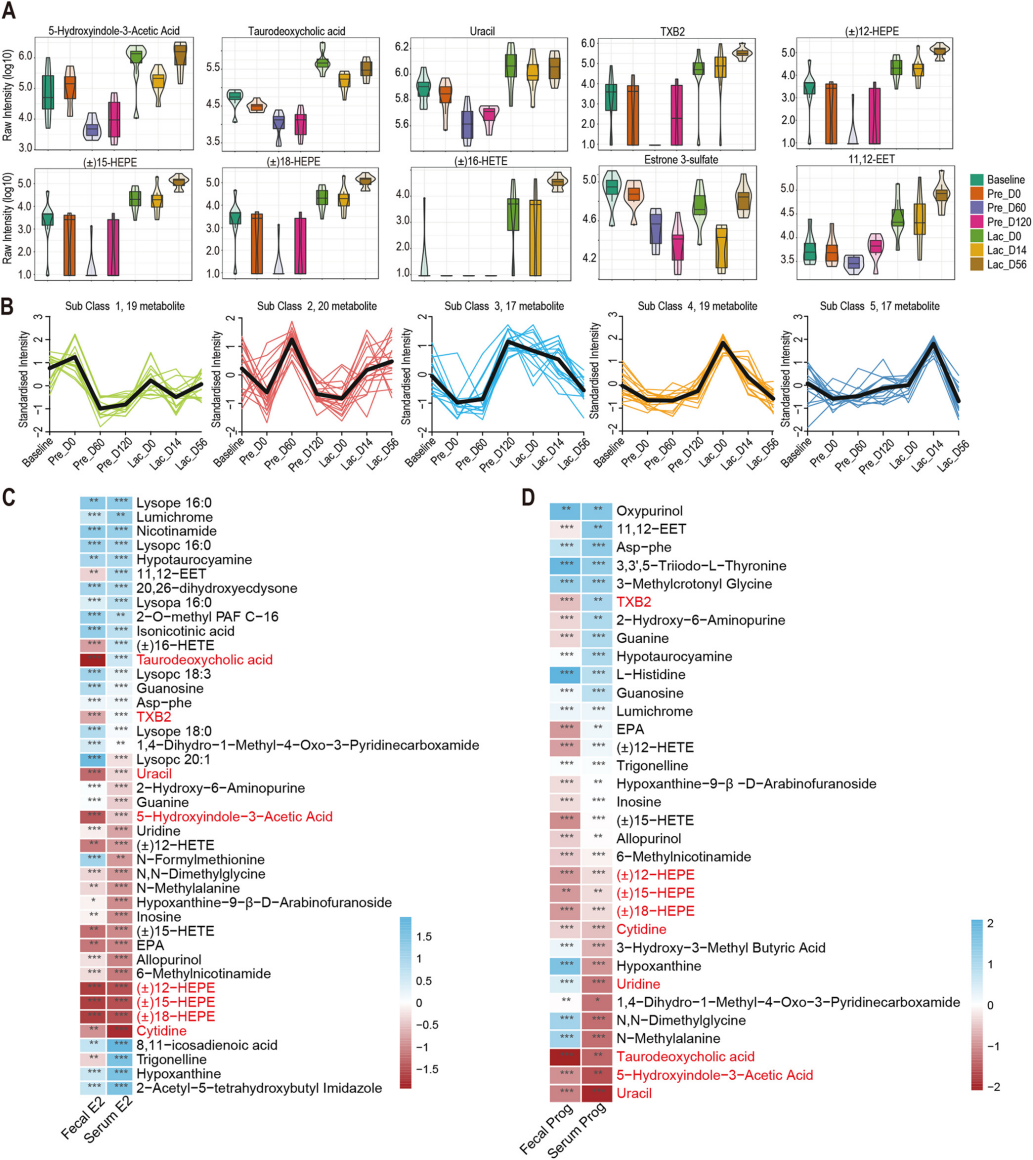
Correlations between the serum metabolites and the concentration of serum parameters during pregnancy and lactation. (A) Relative abundance of significantly different metabolites in goat serum at different stages of pregnancy and lactation. (B) The *K*-means clusters of different metabolites at different stages of pregnancy and lactation. (C) Heatmap correlations between serum metabolites and E_2_ of feces and serum. Color denotes positive (blue) and negative (red) correlation values. |*r*| ≥ 0.4; *, *P < *0.05; **, *P ≤ *0.01; ***, *P ≤ *0.001. (D) Heatmap correlations between serum metabolites and P of feces and serum. Color denotes positive (blue) and negative (red) correlation values. |*r*| ≥ 0.4; *, *P < *0.05; **, *P ≤ *0.01; ***, *P ≤ *0.001.

Comparisons between the metabolic changes in goat serum metabolome from Baseline to Lac_D56 (*n *= 8) by liquid chromatography-mass spectrometry (LC-MS) revealed 563 metabolic features across the different stages. Based on the orthogonal partial least-squares discrimination analysis (OPLS-DA) model, the metabolic features across the different stages of pregnancy and lactation had useful aggregation with an obvious distinction between them (Fig. S5A). Based on the heatmap, the differentially detected metabolites during pregnancy clustered separately from those at Baseline and Lac_D56 (Fig. S5B). The changes in specific metabolites during pregnancy and lactation were assessed to identify the serum metabolome. The overall changes of metabolite are listed in Fig. S5C. Specifically, the abundances of 5-hydroxyindole-3-acetic acid, taurodeoxycholic acid, uracil, thromboxane B2 (TXB2), (±)12-HEPE [(±)-12-hydroxy-5Z,8Z,10E,14Z,17Z-eicosapentaenoic acid], (±)15-HEPE [(±)-15-hydroxy-5Z,8Z,11Z,13E,17Z-eicosapentaenoic acid], (±)18-HEPE [(±)-18-hydroxy-5Z,8Z,11Z,14Z,16E-eicosapentaenoic acid], estrone 3-sulfate, and 11,12-EET [(±)11,(12)-epoxy-5Z,8Z,14Z-eicosatrienoic acid] were significantly decreased at Pre_D60 and Pre_D120 compared to Baseline (Fig. 6A). Intriguingly, (±)16-HETE (5Z,8Z,11Z,14Z,16R-16-hydroxy-5,8,11,14-icosatetraenoic acid) was only detected during the gestation stage of goats (Fig. 6A). Overall, 19, 20, 17, 19, and 17 metabolites were increased at each sampling point, respectively (Fig. 6B; Table S4). The Spearman correlation network analysis further revealed that oxidized lipids, including (±)12-HEPE, (±)15-HEPE, (±)18-HEPE, cytidine, uracil, and 5-hydroxyindole-3-acetic acid, were negatively correlated with the E2 and Prog concentrations of feces and serum samples (*r* < −0.4, *P < *0.05; Fig. 6C and D). Equally important, the abundance of TXB2 was positively correlated with the E2 and Prog concentrations of the serum (*r *> 0.4, *P < *0.05; Fig. 6C and D). The abundance of taurodeoxycholic acid was positively correlated with the E2 and negatively correlated with the Prog concentrations of the serum (*r *> 0.4, *P < *0.05; Fig. 6C and D).

**FIG 7:**
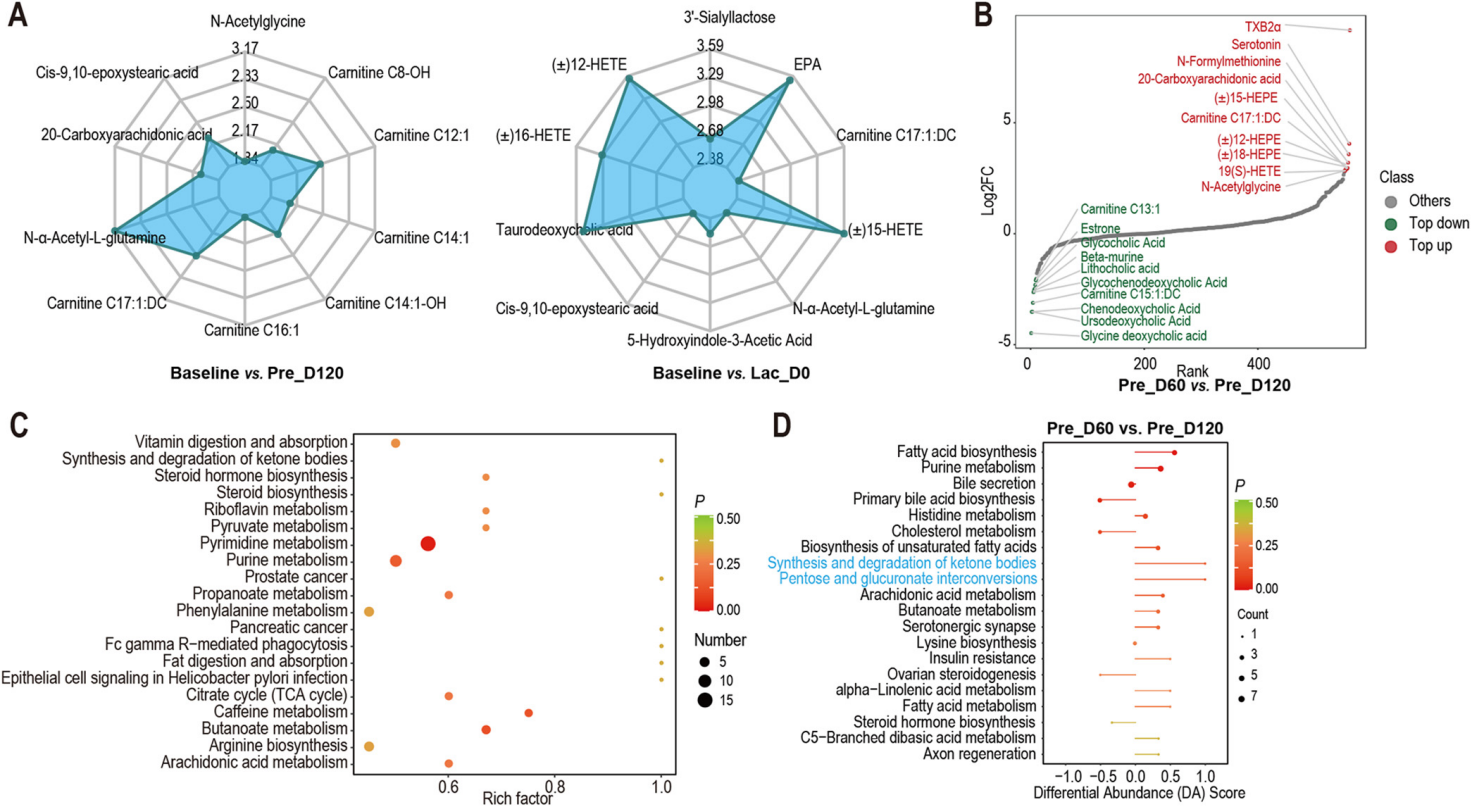
The serum metabolites altered during pregnancy and lactation in goats. (A) The radar chart of differentially expressed metabolites in serum at Baseline versus Pre_D120 and Baseline versus Lac_D0; *n *= 8 individuals/group. (B) The dynamic distribution of differentially expressed metabolites in serum at Pre_D60 versus Pre_D120; *n *= 8 individuals/group. (C) Metabolic pathways are significantly altered during pregnancy and lactation. Red dots denote pregnancy-related pathways at *P ≤ *0.05. (D) Differential abundance (DA) score analysis of KEGG pathway at Pre_D60 versus Pre_D120.

The variable importance in projection ≥1 and absolute log_2_ fold change (FC) ≥1 further revealed that *N*-α-acetyl-l-glutamine, carnitine C8-OH, carnitine C12:1, carnitine C14:1, carnitine C14:1-OH, carnitine C16:1, carnitine C17:1:DC, 20-carboxyarachidonic acid, and cis-9,10-epoxystearic acid were significantly enriched at Pre_D120 (Fig. 7A). (±)12-HETE, (±)16-HETE, taurodeoxycholic acid, cis-9,10-epoxystearic acid, 5-hydroxyindole-3-acetic acid, *N*-α-acetyl-l-glutamine, (±)15-HETE, carnitine C17:1:DC, and EPA (5Z,8Z,11Z,14Z,17Z-eicosapentaenoic acid) were significantly enriched at during Lac_D0 (Fig. 7A) compared to Baseline. In addition, TXB2α (9α,11,15*S*-trihydroxythromba-5Z,13E-dien-1-oic acid), serotonin, *N*-formylmethionine, 20-carboxyarachidonic acid, and (±)15-HEPE were significantly increased in the late trimester (Pre_D120) compared to the first trimester (Pre_D60), while glycine deoxycholic acid, ursodeoxycholic acid, chenodeoxycholic acid, glycochenodeoxycholic acid, and carnitine C15:1:DC were significantly decreased in the late trimester compared to the first trimester (Fig. 7B). In addition, the different metabolites were mainly enriched in pyrimidine (ko00240), butanoate (ko00650), and caffeine metabolism (ko00232) (Table S6; Fig. 7C). Further, KEGG pathway-based analysis of metabolites at DA scores found that the synthesis and degradation of ketone bodies (ko00072) and pentose and glucuronate interconversions (ko00040) were upregulated at the third trimester of pregnancy (Pre_D120) (DA score = 1) compared to the first trimester of pregnancy (Pre_D60) (Fig. 7D).

**FIG 8:**
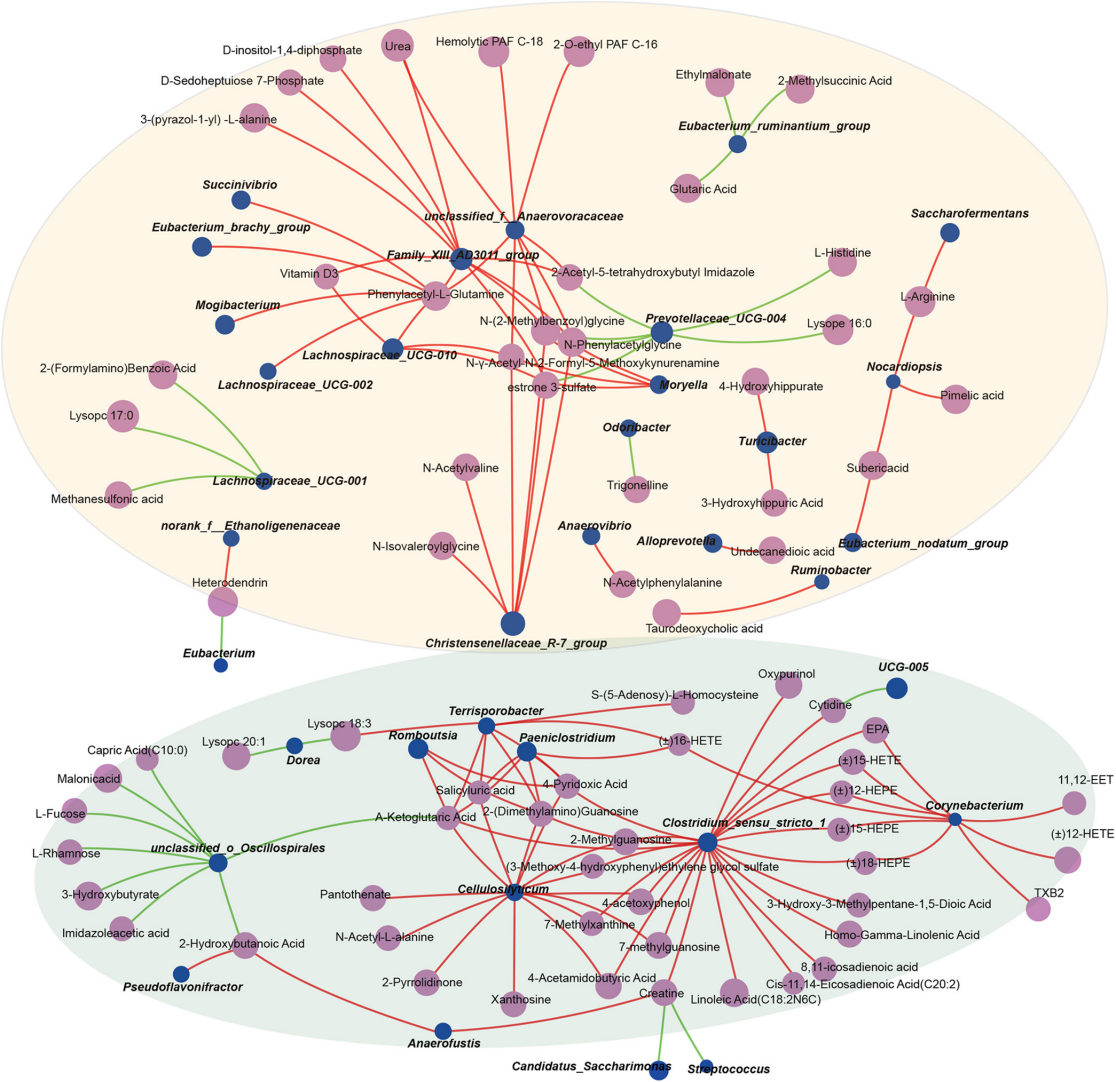
Spearman’s correlation network analysis between the gut microbes and serum metabolome. The line colors ranged from green (negative correlation) to red (positive correlation) at |*r*| ≥ 0.5 and *P < *0.05 level of significance.

The Spearman network correlation analysis was used to evaluate the potential link between alterations in gut microbiota composition and the different serum metabolites. In the serum, it was revealed that *Christensenellaceae_R-7_group*, *Lachnospiraceae_UCG-010*, and *Family_XIII_AD3011_group* were positively correlated with the levels of estrone 3-sulfate (*r *> 0.5, *P < *0.001; Fig. 8), and *Prevotellaceae_UCG-004* was negatively correlated with the levels of Estrone 3-sulfate (*r* < −0.5, *P < *0.001; Fig. 8). In addition, *Corynebacterium* and *Clostridium_sensu_stricto_1* were positively correlated with the abundance of oxidized lipids, including (±)15-HETE, (±)12-HEPE, (±)15-HEPE, and (±)18-HEPE (*r *> 0.5, *P < *0.01; Fig. 8). *Corynebacterium* was also positively correlated with the abundance of 11,12-EET, (±)12-HETE, TXB2, and EPA (*r *> 0.5, *P < *0.001; Fig. 8). We also found *Ruminobacter* was positively correlated with the abundance of taurodeoxycholic acid (*r *> 0.5, *P < *0.001; Fig. 8). Our findings revealed that strong correlations exist between the targeted gut microbial *Family XIII AD3011 group*, *Corynebacterium*, and dramatic alterations in the serum oxidized lipid and hormone metabolite profiles throughout pregnancy and lactation.

## DISCUSSION

Goat physiology undergoes significant changes during nonpregnancy, gestation, and lactation stages. To rule out technical bias and to increase the resolution of changes associated with animals’ physiological status, the diet in the present study was strictly standardized throughout the study. We recognized that goats had increased serum proinflammatory cytokine concentrations and decreased complement and immune cytokine concentrations. Additionally, dramatic changes in serum and intestinal reproductive hormones during gestation, accompanied by decreased serum cholesterol and triglyceride concentrations, were also observed. This implies that reproductive hormone disruptions, maternal inflammation, and lipid metabolism are disordered during gestation, which is consistent with earlier research in other mammals (7, 8, 30, 31). The composition of the maternal gut microbiota changes significantly during pregnancy and lactation in addition to hormonal, immune, and metabolic changes, according to earlier research in monogastric animals (7, 29). Some genera linked to increased insulin sensitivity during pregnancy, such as *Akkermansia* and *Bifidobacteriaceae*, were shown to respond in a previous study in human model (13). To the best of our knowledge, this is the first study to report the dynamic succession pattern of gut microbial alterations and metabolite profiles during the whole reproductive cycle in ruminant species. We investigated two genera that are closely related to E2 and Prog changes, including *Family_XIII AD3011_group* and *Corynebacterium*, in addition to five genera that are closely related to changes in IL-6, particularly *Clostridium_sensu_stricto_1*, *Turicibacter*, *Paeniclostridium*, and *Romboutsia*. These three genera responded to decreased IL-6, and *UCG-005* closely responded to increased IL-6.

Disorders of glucose metabolism, lipid metabolism, and purine metabolism are the main features of the metabolic syndrome during pregnancy. In particular, disorders of purine metabolism lead to increased levels of uric acid in maternal blood (32). In the hindgut of pregnant goats, we detected a high abundance of adenine and purine metabolites, mainly deoxyadenosine, 2′-deoxyinosine, and 5′-deoxyadenosine, which were negatively correlated with the abundance of the genus *Family_XIII_AD3011_group*. Previous studies have shown that intestinal microbiota can be involved in purine metabolism in the body, and in patients with high uric acidity, the abundance of Bacteroides caccae and Bacteroides xylanisolvens was significantly increased, and the levels of *Prevotella* spp, *Ruminococcus* spp, and *Lactobacillus* spp were decreased (33). Escherichia coli, *Lactobacillus*, and Pseudomonas can secrete xanthine dehydrogenase, an enzyme in the oxidative metabolism of purines, responsible for the conversion of xanthine into uric acid (34, 35). In the present study, we could not find direct evidence that the genus *Family_XIII_AD3011_group* bacteria is related to endogenous purine metabolism; hence, the nature of the correlation between the bacteria and fecal metabolites originating from purine metabolism needs further research.

A previous study showed that maternal gut microbes transplanted to germfree mice in late gestation caused obesity and insulin resistance (21). Further research revealed that the ecology of the gut during early and late gestation is completely different and that these microbiome characteristics are frequently linked to phenotypes of the metabolic syndrome (36). In line with the findings of the current study, the maternal immune system is modulated by changes in reproductive hormone concentrations as pregnancy progresses (37), and the secretion of cytokines and other immune substances from placental immune cells and the fetus in pregnant mothers increases local and systemic inflammation. This leads to increased serum concentrations of proinflammatory cytokines and decreased concentrations of immune globulin (37). In addition, certain commensal bacteria in the gut create the precursor neurotransmitter 5-HT as well as neurotransmitters, including dopamine, γ-aminobutyric acid, and norepinephrine (38). According to the findings of the current investigation, dopamine and γ-aminobutyric acid concentrations in the maternal gut decreased in mid to late pregnancy with changes in the composition of gut bacteria. In contrast, E2 and Prog can also modulate the concentration of γ-aminobutyric acid (39), which is an important inhibitory neurotransmitter in the central nervous system. Its abundance has been shown to significantly decrease during gestation, which affects intestinal motility, gastric emptying, sensation, and acid secretion (23, 40). However, further studies are needed to investigate the mechanisms by which the intestinal secretion γ-aminobutyric acid regulates E2 and Prog levels of the host. The present study revealed many acylcarnitine species that are highly elevated during late gestation in goats, including carnitine C12:1, carnitine C17:1:DC, and carnitine C14:1-OH. Changes in acylcarnitine levels in the blood have been linked to polycystic ovarian syndrome (41) and pregnancy (42). Carnitine and acylcarnitine are primarily used in the mitochondria for fatty acid transport and oxidation (43). Fatty acid oxidation and carnitine synthesis happen more quickly in late gestation than in the nonpregnant condition as a result of the rapidly developing fetus (44). To support fetal development and fatty acid oxidation in the placental-fetal unit, the rapidly growing fetus also needs carnitine (45). This requirement is assumed to be predominantly satisfied by placental carnitine absorption from the maternal circulation (46). However, studies on the function of acylcarnitine during late gestation are still in their infancy, so there is a great need for future studies to use metabolomics techniques to understand changes in maternal metabolism in response to different dietary intakes and hormonal changes during pregnancy, as well as the association of metabolite concentrations with fetal and neonatal phenotypes.

Importantly, serum levels of hydroxy eicosapentaenoic acid (HEPE) and hydroxy icosatetraenoic acid (HETE) were markedly increased in goats postpartum, and various metabolites were predominantly enriched in the arachidonic acid metabolic pathway. Previous studies found that arachidonic acid can produce prostaglandins and thromboxanes via cyclooxygenases (47) and arachidonic acid can form hydroxyl derivatives, mainly leukotriene, HPTE, and HETE, in the presence of lipoxygenases (48). Pregnancy and parturition processes are closely linked to the development of physiological inflammatory responses, and parturition leads to changes in the synthesis of inflammatory mediators such as prostaglandins and leukotriene (49), as well as an activation of the cyclooxygenase and 5-lipoxygenase pathways (50). The activation of the cyclooxygenase and 5-lipoxygenase pathways for arachidonic acid conversion promotes increased HPTE and HETE concentrations (50). Regarding the functional role of HPTE and HETE, prior studies in humans have demonstrated that 12-HETE directly contributes to platelet aggregation, cancer, and diabetes (51). 12-HETE and 15-HETE also activate capsaicin-sensitive vanilloid receptors (VR1), which are linked to pain signaling in inflammatory states (52). Additionally, it has been discovered that elevated maternal progesterone levels induce 12/15-lipoxygenase pathway expression, increasing 12/15-lipoxygenase activity and locally increasing concentrations of arachidonic acids (such as 12-HETE and 15-HETE) in the organism (53, 54). These findings suggest that 12-HETE and other metabolites of the lipoxygenase pathway may be involved in regulating placental progesterone biosynthesis (53). Lipoxygenin is produced from linolenic acid when the 15-lipoxygenase pathway is activated. During this period of pregnancy and delivery, lipoxygenins are necessary because they permit vascular remodeling and early placental abruption (53). Additionally, the primary by-products of arachidonic acid metabolism in intrauterine tissues are 5-lipoxygenase and 12-lipoxygenase. The placenta produces lipoxygenase pathway metabolites that may have an impact on myometrial contraction (50). Arachidonic acid may control the smooth muscle tissue’s ability to contract. In comparison to the placenta, the myometrium contains more 15-HETE (50). Once again this demonstrates how hormone-like functions are carried out by arachidonic acid metabolites during both pregnancy and birth. Higher blood levels of 12-HETE and 15-HETE have been linked to pregnancy in a study using cattle as a model (55), and higher serum 12-HETE causes fetal membrane shedding in postpartum cows (56). Interestingly, earlier studies have suggested that HETE is also involved in processes, including vascular tone modulation, renal function, and angiogenesis (57). It was also shown that HETE causes vasodilation and may have anti-inflammatory effects by suppressing NF-κB and vascular smooth muscle cell proliferation (58, 59); hence, a rise in HETE product appears to have a major immunomodulatory impact during late gestation and after parturition. In our study, the abundance of *Corynebacterium* and *Clostridium_sensu_stricto_1* was significantly and positively correlated with the concentrations of HETE and HEPE. However, at present, we did not find direct evidence of their correlation, and further studies are needed at a later stage.

### Conclusions.

Our results showed a specific ecological succession in the gut microbiota during nonpregnancy, pregnancy, and lactation in goats, leading to a decrease in *Family_XIII_AD3011_group* during pregnancy. Combining untargeted metabolomics revealed the landscape of metabolome changes in feces and serum during pregnancy and lactation. Key metabolites of feces and serum were identified, and their levels were associated with hormonal changes. These data can serve as a resource for future research. Overall, the gut microbiota alters the maternal metabolic characteristics and modulates the susceptibility to hormonal changes during pregnancy and lactation and vice versa. However, more research is required to fully understand the specific implications of the target microbes and to develop pharmacological strategies to maintain animal health during pregnancy and lactation.

## MATERIALS AND METHODS

### Ethics statement.

All experimental animal procedures were approved by the Institutional Animal Care and Use Committee of Northwest A&F University (permit number 2019XZ0810050).

### Animals and dietary management.

Before the beginning of this study, 80 Tibet cashmere female goats were tested for estrus by allowing visual and olfactory contact with an intact male goat, and a total of 50 female goats were selected based on the number of deliveries (2 to 3 times). These 50 goats (aged 24 months; average life weight, 26.63 kg) were raised and maintained under the same conditions at Lhasa Tibet Cashmere Goat Breeding Farm (Lhasa, China). The selected goats had no diarrhea or other digestive disorders before and during the study. In addition, they were not put under any medication, including antibiotics and probiotics, during the study. The dietary and nutrient composition fed to the goats during the study is provided in Table S1. The goats had free access to water and feed, and the diet was not changed throughout the study period.

### Experimental design.

The feces and serum samples of baseline (*n *= 50) were collected 5 days while they were not in estrus and immediately before administering a norgestrel-releasing intravaginal device (NRID; 60 mg Prog) to achieve estrus synchronization (NWAFU, Yangling, China). The intravaginal sponge was inserted in the vagina for 14 days, and a total of 400 U pregnant mare serum gonadotropin (PMSG) was administered by intramuscular injection after NRID removal, following our previously published detailed treatment protocol (60). The day after artificial insemination (D0 of gestation; *n *= 50) feces and serum samples were collected from each goat. Pregnancy was detected 45 days after artificial insemination using a B-ultrasonoscope (BXL-V50; Boxianglai, Zhengzhou, China). Subsequently, 18 pregnant goats were selected for feces and serum collection at 60 days (Pre_D60) and 120 days (Pre_D120) of gestation and 0 days (Lac_D0), 14 days (Lac_D14), and 56 days (Lac_D56) of lactation. Lac_D0 samples were taken within 20 min postpartum. The goat kids were housed with their mothers from 0 to 60 days after birth. All goats used in the study were from a singleton pregnancy. A detailed sampling plan is presented in Fig. 1A. To ensure consistency in the number of samples in the study cohort, the nonpregnant goat samples collected at the baseline and Pre_D0 were eliminated and not used in the study. All the feces samples were collected directly from the rectum. The collected feces samples were immediately snap frozen in liquid nitrogen and stored at –80°C, awaiting further analysis. Blood samples (10 mL) were collected from neck veins in centrifugal tubes, and serum samples were collected by centrifuging at 3,000 × *g* for 10 min at 4°C, divided into two samples, put in frozen pipes, and stored at –20°C, awaiting further analysis.

### The serum physiological parameters.

The levels of serum total cholesterol (TC), triglycerides (TG), and glucose (Glu) were measured using the HY-50061, HY-50062, and HY-50063 kits (Huaying, Beijing, China) on an automatic biochemical analyzer (Hitachi 7160, Tokyo, Japan). High-density lipoprotein (HDL) and low-density lipoprotein (LDL) were measured by a colorimetric method using the HY-50070 and HY-50071 kits (Huaying, Beijing, China). Complement 3 (C3) and complement 4 (C4) were measured using the HY-759 kit (Huaying, Beijing, China) on an automatic biochemical analyzer (Hitachi7160, Tokyo, Japan). Serum immunoglobulin A (IgA), immunoglobulin G (IgG), and immunoglobulin M (IgM) were measured by the sandwich ELISA method using the IgG/A/M (HY-50094) kit (Huaying, Beijing, China). Serum levels of IL-6, IL-10, TNF-α, and IFN-γ were measured using ELISA kit in accordance with the manufacturer’s instructions (Huaying, Beijing, China). Serum E2 was measured using the HY-10029 IRMA kit, Prog was measured using the HY-10028 RIA kit (Huaying, Beijing, China), and 5-HT was measured using the HY-10200 RIA kit (Huaying, Beijing, China) in accordance with the manufacturer’s instructions.

### Fecal estradiol and progesterone concentration.

A total of 70 (*n *= 10) fecal samples were dried in a freeze dryer for 24 h and then 1.6 mL of 80% ethanol was added to the dried samples. The samples were vortexed for 30 s and incubated in a 70°C water bath for 20 min. The obtained suspension was centrifuged at 3,000 × *g* for 15 min, and the supernatant was collected. The above-described process was repeated with the sediments. The two supernatants were pooled and heated in a 70°C water bath until the liquid fraction was completely evaporated. Subsequently, 500 μL methanol was added to each sample. The dissolved extracts were transferred into centrifuge tubes (61), snap frozen, and stored at –20°C, awaiting further analysis. The E2 and Prog concentrations in the feces were measured using the HY-10029 IRMA and HY-10028 RIA kits (Huaying, Beijing, China) following the manufacturer’s instructions.

### DNA Extraction, 16S rRNA Gene Amplification, and Illumina MiSeq Sequencing.

The 126 fecal samples (*n *= 18) were subjected to DNA extraction using the E.Z.N.A. stool DNA kit (Omega Bio-tek, Norcross, GA, USA) according to the manufacturer’s protocol. The extracted DNA concentration and purity were determined using the Nanodrop 2000 UV-VI spectrophotometer (Thermo Scientific, Wilmington, DE, USA), while the quality was assessed using 1% agarose gel electrophoresis. The DNA was then amplified using the primers 338F (5′-ACTCCTACGGGAGGCAGCAG-3′) and 806R (5′-GGACTACHVGGGTWTCTAAT-3′) targeting the V3 to V4 16S rRNA gene regions on a thermocycler PCR system (TransGen AP221-02, TransGen Biotech, China). PCR was performed using the TransGen kit (TransGen AP221-02; TransStart FastPfu DNA polymerase, TransGen Biotech, Beijing, China) following the manufacturer’s guidelines (62). Finally, the purified amplicons were pooled into equimolar ratios and subjected to paired-end sequencing (2 × 300 bp) on an Illumina MiSeq platform (Illumina, San Diego, CA, USA) at the Major Biobio-Pharm Technology Co., Ltd. (Major Biobio, Shanghai, China).

### Microbiome data analysis.

The Illumina (fastq) data were imported into the QIIME2 platform (version 2020.2) (63) and analyzed on the online platform of the Majorbio Cloud Platform (64). Briefly, the sequences were demultiplexed, followed by merging the pair-ended reads using the FLASH software (version 1.2.11) (65) and low-quality reads were filtered using the FASTP software (version 0.19.6) (66). Next, the sequences were filtered, denoised, and merged, and chimeras were removed using the DADA2 plugin (67) in the QIIME2 platform. The number of sequences per sample was rarefied to 23,400 to minimize the effects of sequencing depth on alpha and beta diversity measures, achieving an average Good’s coverage of 99.83%. Taxonomic assignment of ASVs was performed using the naive Bayes consensus taxonomy classifier implemented in QIIME2 against the SILVA 138/16s_bacteria.

### Untargeted metabolomics using UHPLC-ESI-MS/MS.

Total 56 fecal samples (*n *= 8) were thawed on ice as part of the sample preparation and extraction process. A total of 50 mg (±1 mg) of each sample was taken and homogenized with 500 μL of ice-cold methanol/water (70%, vol/vol). The samples were vortexed for 3 min, sonicated for 10 min in an ice water bath, and then vortexed again for 1 min. The samples were centrifuged (12,000 rpm) at 4°C for 10 min. A total of 250 μL of the supernatant was taken to the centrifuge tube, and the supernatant was centrifuged (12,000 rpm) at 4°C for 5 min. Then, 150 μL of the supernatant in the liner of the corresponding injection bottle was taken for onboard analysis. The following steps were taken to prepare the serum samples: A total of 56 serum samples (*n *= 8) were thawed on ice, vortexed for 10 s, and mix well. A total of 300 μL of pure methanol was added to 50 μL of serum, and then the mixture was whirled for 3 min and centrifuged (12,000 rpm) at 4°C for 10 min. The supernatant was collected and centrifuged (12,000 rpm) at 4°C for 5 min. The samples were left in a refrigerator at –20°C for 30 min, centrifuged (12,000 rpm) at 4°C for 3 min, and 150 μL of supernatant in the liner of the corresponding injection bottle was taken for onboard analysis. The feces and serum sample extracts were analyzed using an UHPLC-ESI-MS/MS system (ultra-high-performance liquid chromatography [UHPLC], ExionLC AD; electrospray ionization [ESI]; tandem mass spectrometry [MS], QTRAP System, Boston, MA, USA). The analytical conditions were as follows, UHPLC: column, Waters ACQUITY UPLC HSS T3 C18 (1.8 μm, 2.1 mm × 100 mm); column temperature, 40°C; flow rate, 0.4 mL/min; injection volume, 2 μL; solvent system: solvent A was water with 0.1% formic acid and solvent B was acetonitrile with 0.1% formic acid; gradient program, 95:5 vol/vol at 0 min, 10:90 vol/vol at 10.0 min, 10:90 vol/vol at 11.0 min, 95:5 vol/V at 11.1 min, and 95:5 vol/vol at 14.0 min (68). Linear ion trap (LIT) and triple quadrupole (QQQ) scans were acquired on a triple quadrupole-linear ion trap mass spectrometer (QTRAP), the QTRAP LC-MS/MS System, equipped with an ESI Turbo Ion-Spray interface, operated in positive and negative ion mode, and controlled by Analyst 1.6.3 software (68). The ESI source operation parameters were as follows: source temperature 500°C; ion spray voltage (IS), 5,500 V (positive) and −4,500 V (negative); ion source gas I (GSI), gas II (GSII), and curtain gas (CUR) were set at 55, 60, and 25.0 lb/in^2^, respectively; the collision gas was set as “high” (12 lb/in^2^). Instrument tuning and mass calibration were performed with 10 to 100 μmol/L polypropylene glycol solutions in QQQ and LIT modes, respectively. In detail, test experiments were performed with 10 to 100 μmol/L polypropylene glycol solutions in LIT or QQQ mode under the instrument to confirm that the instrument mass axis and signal response are normal and can be used for sample detection and analysis. The dwell time was in the range of 3 to 50 ms. The collision energy (CE) ranges from 5 to 50 for positive ion mode and −5 to 50 for negative ion mode. Depending on the retention time of the compound, the residence time of each multiple reaction monitoring (MRM) ion channel was in the range of 3 to 50 ms, and the total scan time was 1.2 s. A specific set of MRM transitions were monitored for each period according to the metabolites eluted during this period. The LC-MS/MS data were processed by Analyst 1.6.3 software package (68). Based on the self-built MWDB (METWARE database; Metware Biotechnology Co., Ltd., Wuhan, China), including 826 metabolomic features. The mass spectrometry file of each sample was integrated with MultiQuant software, and the peak area of each peak represented the relative content of the corresponding substance. The peak areas detected for each MRM ion pair in all samples were corrected using MultiQuant software so that their integration parameters (retention time, peak width, and baseline) were consistent. Finally, all the chromatographic peak area integration data were exported and saved (69). We provide detailed results information of the fecal and serum metabolomic data in Table S2 and Table S3, respectively. Significantly regulated metabolites between groups were determined by VIP >1 and absolute log_2_ FC ≥1. The identified metabolites were annotated using the KEGG compound database, and the annotated metabolites were mapped to the KEGG pathway database. Significantly enriched pathways were identified using the hypergeometric test.

### Statistical methods.

All data were presented as mean ± SEM at *, *P < *0.05, **, *P ≤ *0.01, and ***, *P ≤ *0.001. Differences of serum and fecal hormone data were compared using one-way ANOVA. The linear fitting and normalization were performed using the GraphPad Prism (V8.3) software. In addition, the changes in taxonomic community composition and the relative abundance in all the microbial species were tested using the nonparametric Kruskal–Wallis tests followed by the Tukey-Kramer post hoc test using a Benjamin-Holmes false discovery rate (FDR) correction. The alpha diversity index under different random samples was calculated using Mothur-1.30 (70) and beta-diversity (PCoA based on Bray-Curtis dissimilarities) with the R-3.3.1 (vegan) package. Alpha diversity (Chao) was compared by an ANOVA model splitting the variation into each group. For PCoA analysis, the ANOSIM function in the vegan package in R was used, including different independent variables based on 999 permutations. The bar figure was calculated using the python-2.7 package. The R-3.3.1 (stat) was used to perform the network correlation analysis. Spearman’s correlation coefficient was calculated in R using cor with method = set to “spearman.” Factors were added to the cooccurrence network if the absolute value of the correlation coefficient was greater than 0.5. For significantly altered compound analyses of two group, distribution-independent ranking tests (based on the Wilcoxon test) and the sample-wise permutation (default by thesamr package) were used to ascertain significance (FDR < 0.05) (71). VIP values extracted from the OPLS-DA result, which also contain score plots and permutation plots, were generated using the R package MetaboAnalyst R (72). *K*-means clustering was performed in R. Data were checked for normality using the D’Agostino-Pearson omnibus test (73). The identified metabolites were annotated in the KEGG compound database before being linked to the KEGG pathway database (74).

### Data availability.

The 16S rRNA gene sequencing data are available from the national center for biotechnology information (NCBI) under accession no. PRJNA786102.

## References

[1] Kumar P, Magon N. 2012. Hormones in pregnancy. Niger Med J 53:179–183. doi:10.4103/0300-1652.107549. 23661874 PMC3640235

[2] Pethick D, Lindsay D, Barker PJ, Northrop A. 1983. The metabolism of circulating non-esterified fatty acids by the whole animal, hind-limb muscle and uterus of pregnant ewes. Br J Nutr 49:129–143. doi:10.1079/bjn19830018. 6821682

[3] Newbern D, Freemark M. 2011. Placental hormones and the control of maternal metabolism and fetal growth. Curr Opin Endocrinol Diabetes Obes 18:409–416. doi:10.1097/MED.0b013e32834c800d. 21986512

[4] Mosnier E, Le Floc'H N, Etienne M, Ramaekers P, Sève B, Père M-C. 2010. Reduced feed intake of lactating primiparous sows is associated with increased insulin resistance during the peripartum period and is not modified through supplementation with dietary tryptophan. J Anim Sci 88:612–625. doi:10.2527/jas.2008-1768. 19855001

[5] Chatterjee P, Chiasson VL, Bounds KR, Mitchell BM. 2014. Regulation of the anti-inflammatory cytokines interleukin-4 and interleukin-10 during pregnancy. Front Immunol 5:253. doi:10.3389/fimmu.2014.00253. 24904596 PMC4034149

[6] Tan C, Wei H, Sun H, Ao J, Long G, Jiang S, Peng J. 2015. Effects of dietary supplementation of oregano essential oil to sows on oxidative stress status, lactation feed intake of sows, and piglet performance. Biomed Res Int 2015:525218. doi:10.1155/2015/525218. 26539506 PMC4619846

[7] Liu H, Hou C, Li N, Zhang X, Zhang G, Yang F, Zeng X, Liu Z, Qiao S. 2019. Microbial and metabolic alterations in gut microbiota of sows during pregnancy and lactation. FASEB J 33:4490–4501. doi:10.1096/fj.201801221RR. 30653349

[8] Zheng W, Xu Q, Huang W, Yan Q, Chen Y, Zhang L, Tian Z, Liu T, Yuan X, Liu C. 2020. Gestational diabetes mellitus is associated with reduced dynamics of gut microbiota during the first half of pregnancy. Msystems 5:e00109-20. doi:10.1128/mSystems.00109-20. 32209715 PMC7093821

[9] Xue Y, Lin L, Hu F, Zhu W, Mao S. 2020. Disruption of ruminal homeostasis by malnutrition involved in systemic ruminal microbiota-host interactions in a pregnant sheep model. Microbiome 8:1–14. doi:10.1186/s40168-020-00916-8. 32972462 PMC7517653

[10] Kimura I, Miyamoto J, Ohue-Kitano R, Watanabe K, Yamada T, Onuki M, Aoki R, Isobe Y, Kashihara D, Inoue D. 2020. Maternal gut microbiota in pregnancy influences offspring metabolic phenotype in mice. Science 367:eaaw8429. doi:10.1126/science.aaw8429. 32108090

[11] Nyangahu DD, Lennard KS, Brown BP, Darby MG, Wendoh JM, Havyarimana E, Smith P, Butcher J, Stintzi A, Mulder N. 2018. Disruption of maternal gut microbiota during gestation alters offspring microbiota and immunity. Microbiome 6:124. doi:10.1186/s40168-018-0511-7. 29981583 PMC6035804

[12] Kerr CA, Grice DM, Tran CD, Bauer DC, Li D, Hendry P, Hannan GN. 2015. Early life events influence whole-of-life metabolic health via gut microflora and gut permeability. Crit Rev Microbiol 41:326–340. doi:10.3109/1040841X.2013.837863. 24645635

[13] Gohir W, Whelan FJ, Surette MG, Moore C, Schertzer JD, Sloboda DM. 2015. Pregnancy-related changes in the maternal gut microbiota are dependent upon the mother's periconceptional diet. Gut Microbes 6:310–320. doi:10.1080/19490976.2015.1086056. 26322500 PMC4826136

[14] Shen J, Obin MS, Zhao L. 2013. The gut microbiota, obesity and insulin resistance. Mol Aspects Med 34:39–58. doi:10.1016/j.mam.2012.11.001. 23159341

[15] Mokkala K, Röytiö H, Munukka E, Pietilä S, Ekblad U, Rönnemaa T, Eerola E, Laiho A, Laitinen K. 2016. Gut microbiota richness and composition and dietary intake of overweight pregnant women are related to serum zonulin concentration, a marker for intestinal permeability. J Nutr 146:1694–1700. doi:10.3945/jn.116.235358. 27466607

[16] Lv Y, Yan Z, Zhao X, Gang X, He G, Sun L, Li Z, Wang G. 2018. The effects of gut microbiota on metabolic outcomes in pregnant women and their offspring. Food Funct 9:4537–4547. doi:10.1039/c8fo00601f. 30101246

[17] Ganal-Vonarburg SC, Hornef MW, Macpherson AJ. 2020. Microbial–host molecular exchange and its functional consequences in early mammalian life. Science 368:604–607. doi:10.1126/science.aba0478. 32381716

[18] Neuman H, Debelius JW, Knight R, Koren O. 2015. Microbial endocrinology: the interplay between the microbiota and the endocrine system. FEMS Microbiol Rev 39:509–521. doi:10.1093/femsre/fuu010. 25701044

[19] Nakatani Y, Sato-Suzuki I, Tsujino N, Nakasato A, Seki Y, Fumoto M, Arita H. 2008. Augmented brain 5-HT crosses the blood–brain barrier through the 5-HT transporter in rat. Eur J Neurosci 27:2466–2472. doi:10.1111/j.1460-9568.2008.06201.x. 18445233

[20] Ge X, Ding C, Zhao W, Xu L, Tian H, Gong J, Zhu M, Li J, Li N. 2017. Antibiotics-induced depletion of mice microbiota induces changes in host serotonin biosynthesis and intestinal motility. J Translational Medicine 15:13. doi:10.1186/s12967-016-1105-4. PMC5237163 28086815

[21] Koren O, Goodrich JK, Cullender TC, Spor A, Laitinen K, Bäckhed HK, Gonzalez A, Werner JJ, Angenent LT, Knight R, Bäckhed F, Isolauri E, Salminen S, Ley RE. 2012. Host remodeling of the gut microbiome and metabolic changes during pregnancy. Cell 150:470–480. doi:10.1016/j.cell.2012.07.008. 22863002 PMC3505857

[22] Mayer EA, Savidge T, Shulman RJ. 2014. Brain–gut microbiome interactions and functional bowel disorders. Gastroenterology 146:1500–1512. doi:10.1053/j.gastro.2014.02.037. 24583088 PMC4114504

[23] Strandwitz P. 2018. Neurotransmitter modulation by the gut microbiota. Brain Res 1693:128–133. doi:10.1016/j.brainres.2018.03.015. 29903615 PMC6005194

[24] Baker JM, Al-Nakkash L, Herbst-Kralovetz MM. 2017. Estrogen–gut microbiome axis: physiological and clinical implications. Maturitas 103:45–53. doi:10.1016/j.maturitas.2017.06.025. 28778332

[25] Insenser M, Murri M, Del Campo R, Martinez-Garcia MA, Fernandez-Duran E, Escobar-Morreale HF. 2018. Gut microbiota and the polycystic ovary syndrome: influence of sex, sex hormones, and obesity. J Clin Endocrinol Metab 103:2552–2562. doi:10.1210/jc.2017-02799. 29897462

[26] Brubaker PL. 2018. Linking the gut microbiome to metabolism through endocrine hormones. Endocrinology 159:2978–2979. doi:10.1210/en.2018-00577. 29931261

[27] Markle JG, Frank DN, Mortin-Toth S, Robertson CE, Feazel LM, Rolle-Kampczyk U, Von Bergen M, McCoy KD, Macpherson AJ, Danska JS. 2013. Sex differences in the gut microbiome drive hormone-dependent regulation of autoimmunity. Science 339:1084–1088. doi:10.1126/science.1233521. 23328391

[28] Cheng C, Wei H, Yu H, Xu C, Jiang S, Peng J. 2018. Metabolic syndrome during perinatal period in sows and the link with gut microbiota and metabolites. Front Microbiol 9:1989. doi:10.3389/fmicb.2018.01989. 30197635 PMC6117386

[29] Liang L, Rasmussen M-LH, Piening B, Shen X, Chen S, Röst H, Snyder JK, Tibshirani R, Skotte L, Lee NC, Contrepois K, Feenstra B, Zackriah H, Snyder M, Melbye M. 2020. Metabolic dynamics and prediction of gestational age and time to delivery in pregnant women. Cell 181:1680–1692.e15. doi:10.1016/j.cell.2020.05.002. 32589958 PMC7327522

[30] Xue C, Xie Q, Zhang C, Hu Y, Song X, Jia Y, Shi X, Chen Y, Liu Y, Zhao L, Huang F, Yuan H. 2022. Vertical transmission of the gut microbiota influences glucose metabolism in offspring of mice with hyperglycaemia in pregnancy. Microbiome 10:122. doi:10.1186/s40168-022-01318-8. 35941695 PMC9361546

[31] Wang J, Zheng J, Shi W, Du N, Xu X, Zhang Y, Ji P, Zhang F, Jia Z, Wang Y, Zheng Z, Zhang H, Zhao F. 2018. Dysbiosis of maternal and neonatal microbiota associated with gestational diabetes mellitus. Gut 67:1614–1625. doi:10.1136/gutjnl-2018-315988. 29760169 PMC6109274

[32] Guo Z, Zhang J, Wang Z, Ang KY, Huang S, Hou Q, Su X, Qiao J, Zheng Y, Wang L, Koh E, Danliang H, Xu J, Lee YK, Zhang H. 2016. Intestinal microbiota distinguish gout patients from healthy humans. Sci Rep 6:20602. doi:10.1038/srep20602. 26852926 PMC4757479

[33] Wang Z, Li Y, Liao W, Huang J, Liu Y, Li Z, Tang J. 2022. Gut microbiota remodeling: a promising therapeutic strategy to confront hyperuricemia and gout. Front Cell Infect Microbiol 12:935723. doi:10.3389/fcimb.2022.935723. 36034697 PMC9399429

[34] Wu Y, Ye Z, Feng P, Li R, Chen X, Tian X, Han R, Kakade A, Liu P, Li X. 2021. Limosilactobacillus fermentum JL-3 isolated from “Jiangshui” ameliorates hyperuricemia by degrading uric acid. Gut Microbes 13:1–18. doi:10.1080/19490976.2021.1897211. PMC8007157 33764849

[35] Chu Y, Sun S, Huang Y, Gao Q, Xie X, Wang P, Li J, Liang L, He X, Jiang Y, Wang M, Yang J, Chen X, Zhou C, Zhao Y, Ding F, Zhang Y, Wu X, Bai X, Wu J, Wei X, Chen X, Yue Z, Fang X, Huang Q, Wang Z, Huang R. 2021. Metagenomic analysis revealed the potential role of gut microbiome in gout. NPJ Biofilms Microbiomes 7:66. doi:10.1038/s41522-021-00235-2. 34373464 PMC8352958

[36] Ramos B, Kanninen TT, Sisti G, Witkin SS. 2015. Microorganisms in the female genital tract during pregnancy: tolerance versus pathogenesis. Am J Reprod Immunol 73:383–389. doi:10.1111/aji.12326. 25244611

[37] Mor G, Cardenas I. 2010. The immune system in pregnancy: a unique complexity. Am J Reprod Immunol 63:425–433. doi:10.1111/j.1600-0897.2010.00836.x. 20367629 PMC3025805

[38] Strandwitz P, Kim KH, Terekhova D, Liu JK, Sharma A, Levering J, McDonald D, Dietrich D, Ramadhar TR, Lekbua A, Mroue N, Liston C, Stewart EJ, Dubin MJ, Zengler K, Knight R, Gilbert JA, Clardy J, Lewis K. 2019. GABA-modulating bacteria of the human gut microbiota. Nat Microbiol 4:396–403. doi:10.1038/s41564-018-0307-3. 30531975 PMC6384127

[39] Morrow AL. 2007. Recent developments in the significance and therapeutic relevance of neuroactive steroids—introduction to the special issue. Pharmacol Ther 116:1–6. doi:10.1016/j.pharmthera.2007.04.003. 17531324 PMC2047816

[40] Hyland NP, Cryan JF. 2010. A gut feeling about GABA: focus on GABAB receptors. Frontiers in Pharmacology 1:124. doi:10.3389/fphar.2010.00124. 21833169 PMC3153004

[41] Fenkci SM, Fenkci V, Oztekin O, Rota S, Karagenc N. 2008. Serum total L-carnitine levels in non-obese women with polycystic ovary syndrome. Hum Reprod 23:1602–1606. doi:10.1093/humrep/den109. 18378560

[42] Cederblad G, Fåhraeus L, Lindgren K. 1986. Plasma carnitine and renal-carnitine clearance during pregnancy. Am J Clin Nutr 44:379–383. doi:10.1093/ajcn/44.3.379. 3751959

[43] Houten SM, Wanders RJ. 2010. A general introduction to the biochemistry of mitochondrial fatty acid β-oxidation. J Inherit Metab Dis 33:469–477. doi:10.1007/s10545-010-9061-2. 20195903 PMC2950079

[44] Hadden DR, McLaughlin C. 2009. Normal and abnormal maternal metabolism during pregnancy. Semin Fetal Neonatal Med 14:66–71. doi:10.1016/j.siny.2008.09.004. 18986856

[45] Arenas J, Rubio JC, Martín MA, Campos Y. 1998. Biological roles of L-carnitine in perinatal metabolism. Early Hum Dev 53:S43–S50. doi:10.1016/s0378-3782(98)00064-4. 10102654

[46] Grube M, Zu Schwabedissen HM, Draber K, Präger D, Möritz K-U, Linnemann K, Fusch C, Jedlitschky G, Kroemer HK. 2005. Expression, localization, and function of the carnitine transporter octn2 (slc22a5) in human placenta. Drug Metab Dispos 33:31–37. doi:10.1124/dmd.104.001560. 15486076

[47] Smith WL, Urade Y, Jakobsson P-J. 2011. Enzymes of the cyclooxygenase pathways of prostanoid biosynthesis. Chem Rev 111:5821–5865. doi:10.1021/cr2002992. 21942677 PMC3285496

[48] Gabbs M, Leng S, Devassy JG, Monirujjaman M, Aukema HM. 2015. Advances in our understanding of oxylipins derived from dietary PUFAs. Adv Nutr 6:513–540. doi:10.3945/an.114.007732. 26374175 PMC4561827

[49] Waldman M, Peterson SJ, Arad M, Hochhauser E. 2016. The role of 20-HETE in cardiovascular diseases and its risk factors. Prostaglandins Other Lipid Mediat 125:108–117. doi:10.1016/j.prostaglandins.2016.05.007. 27287720

[50] Kikut J, Komorniak N, Ziętek M, Palma J, Szczuko M. 2020. Inflammation with the participation of arachidonic (AA) and linoleic acid (LA) derivatives (HETEs and HODEs) is necessary in the course of a normal reproductive cycle and pregnancy. J Reprod Immunol 141:103177. doi:10.1016/j.jri.2020.103177. 32659532

[51] Powell WS, Rokach J. 2015. Biosynthesis, biological effects, and receptors of hydroxyeicosatetraenoic acids (HETEs) and oxoeicosatetraenoic acids (oxo-ETEs) derived from arachidonic acid. Biochim Biophys Acta 1851:340–355. doi:10.1016/j.bbalip.2014.10.008. 25449650 PMC5710736

[52] Buczynski MW, Dumlao DS, Dennis EA. 2009. Thematic Review Series: proteomics. An integrated omics analysis of eicosanoid biology1 [S]. J Lipid Res 50:1015–1038. doi:10.1194/jlr.R900004-JLR200. 19244215 PMC2681385

[53] Li Q, Cheon Y-P, Kannan A, Shanker S, Bagchi IC, Bagchi MK. 2004. A novel pathway involving progesterone receptor, 12/15-lipoxygenase-derived eicosanoids, and peroxisome proliferator-activated receptor γ regulates implantation in mice. J Biol Chem 279:11570–11581. doi:10.1074/jbc.M311773200. 14688261

[54] Sato K, Chisaka H, Okamura K, Challis JR. 2008. Effect of the interaction between lipoxygenase pathway and progesterone on the regulation of hydroxysteroid 11-beta dehydrogenase 2 in cultured human term placental trophoblasts. Biol Reprod 78:514–520. doi:10.1095/biolreprod.107.064717. 18032417

[55] Sponchiado M, Gonella-Diaza AM, Rocha CC, Turco EGL, Pugliesi G, Leroy JL, Binelli M. 2019. The pre-hatching bovine embryo transforms the uterine luminal metabolite composition in vivo. Scientific Rep 9:8354. doi:10.1038/s41598-019-44590-9. PMC6555789 31175317

[56] Kamada H, Matsui Y, Sakurai Y, Tanigawa T, Itoh M, Kawamoto S, Kai K, Sasaki T, Takahashi K, Hayashi M, Takayama Y, Nakamura M, Kadokawa H, Ueda Y, Sutoh M, Murai M. 2012. Twelve oxo-eicosatetraenoic acid induces fetal membrane release after delivery in cows. Placenta 33:106–113. doi:10.1016/j.placenta.2011.11.001. 22118869

[57] Jiang H, McGiff JC, Fava C, Amen G, Nesta E, Zanconato G, Quilley J, Minuz P. 2013. Maternal and fetal epoxyeicosatrienoic acids in normotensive and preeclamptic pregnancies. Am J Hypertens 26:271–278. doi:10.1093/ajh/hps011. 23382413 PMC3935001

[58] Elmarakby AA. 2012. Reno-protective mechanisms of epoxyeicosatrienoic acids in cardiovascular disease. Am J Physiol Regul Integr Comp Physiol 302:R321–R330. doi:10.1152/ajpregu.00606.2011. 22116511

[59] Zhang J-H, Pearson T, Matharoo-Ball B, Ortori CA, Warren AY, Khan R, Barrett DA. 2007. Quantitative profiling of epoxyeicosatrienoic, hydroxyeicosatetraenoic, and dihydroxyeicosatetraenoic acids in human intrauterine tissues using liquid chromatography/electrospray ionization tandem mass spectrometry. Anal Biochem 365:40–51. doi:10.1016/j.ab.2007.03.001. 17418798

[60] Wang X, Yu H, Lei A, Zhou J, Zeng W, Zhu H, Dong Z, Niu Y, Shi B, Cai B. 2015. Generation of gene-modified goats targeting MSTN and FGF5 via zygote injection of CRISPR/Cas9 system. Scientific Rep 5:13878. doi:10.1038/srep13878. PMC4564737 26354037

[61] Capezzuto A, Chelini M, Felippe E, Oliveira C. 2008. Correlation between serum and fecal concentrations of reproductive steroids throughout gestation in goats. Anim Reprod Sci 103:78–86. doi:10.1016/j.anireprosci.2006.11.001. 17156948

[62] Li B, Zhang K, Li C, Wang X, Chen Y, Yang Y. 2019. Characterization and comparison of microbiota in the gastrointestinal tracts of the goat (Capra hircus) during preweaning development. Front Microbiol 10:2125. doi:10.3389/fmicb.2019.02125. 31572331 PMC6753876

[63] Bolyen E, Rideout JR, Dillon MR, Bokulich NA, Abnet CC, Al-Ghalith GA, Alexander H, Alm EJ, Arumugam M, Asnicar F, Bai Y, Bisanz JE, Bittinger K, Brejnrod A, Brislawn CJ, Brown CT, Callahan BJ, Caraballo-Rodríguez AM, Chase J, Cope EK, Da Silva R, Diener C, Dorrestein PC, Douglas GM, Durall DM, Duvallet C, Edwardson CF, Ernst M, Estaki M, Fouquier J, Gauglitz JM, Gibbons SM, Gibson DL, Gonzalez A, Gorlick K, Guo J, Hillmann B, Holmes S, Holste H, Huttenhower C, Huttley GA, Janssen S, Jarmusch AK, Jiang L, Kaehler BD, Kang KB, Keefe CR, Keim P, Kelley ST, Knights D, et al. 2019. Reproducible, interactive, scalable and extensible microbiome data science using QIIME 2. Nat Biotechnol 37:852–857. doi:10.1038/s41587-019-0209-9. 31341288 PMC7015180

[64] Zheng Z, Cai Y, Zhang Y, Zhao Y, Gao Y, Cui Z, Hu Y, Wang X. 2021. The effects of C/N (10–25) on the relationship of substrates, metabolites, and microorganisms in “inhibited steady-state” of anaerobic digestion. Water Res 188:116466. doi:10.1016/j.watres.2020.116466. 33027695

[65] Magoč T, Salzberg SL. 2011. FLASH: fast length adjustment of short reads to improve genome assemblies. Bioinformatics 27:2957–2963. doi:10.1093/bioinformatics/btr507. 21903629 PMC3198573

[66] Chen S, Zhou Y, Chen Y, Gu J. 2018. fastp: an ultra-fast all-in-one FASTQ preprocessor. Bioinformatics 34:i884–i890. doi:10.1093/bioinformatics/bty560. 30423086 PMC6129281

[67] Callahan BJ, McMurdie PJ, Rosen MJ, Han AW, Johnson AJA, Holmes SP. 2016. DADA2: high-resolution sample inference from Illumina amplicon data. Nat Methods 13:581–583. doi:10.1038/nmeth.3869. 27214047 PMC4927377

[68] Chen W, Gong L, Guo Z, Wang W, Zhang H, Liu X, Yu S, Xiong L, Luo J. 2013. A novel integrated method for large-scale detection, identification, and quantification of widely targeted metabolites: application in the study of rice metabolomics. Mol Plant 6:1769–1780. doi:10.1093/mp/sst080. 23702596

[69] Fraga CG, Clowers BH, Moore RJ, Zink EM. 2010. Signature-discovery approach for sample matching of a nerve-agent precursor using liquid chromatography−mass spectrometry, XCMS, and chemometrics. Anal Chem 82:4165–4173. doi:10.1021/ac1003568. 20405949

[70] Schloss PD, Westcott SL, Ryabin T, Hall JR, Hartmann M, Hollister EB, Lesniewski RA, Oakley BB, Parks DH, Robinson CJ, Sahl JW, Stres B, Thallinger GG, Van Horn DJ, Weber CF. 2009. Introducing mothur: open-source, platform-independent, community-supported software for describing and comparing microbial communities. Appl Environ Microbiol 75:7537–7541. doi:10.1128/AEM.01541-09. 19801464 PMC2786419

[71] Tusher VG, Tibshirani R, Chu G. 2001. Significance analysis of microarrays applied to the ionizing radiation response. Proc Natl Acad Sci USA 98:5116–5121. doi:10.1073/pnas.091062498. 11309499 PMC33173

[72] Xia J, Psychogios N, Young N, Wishart DS. 2009. MetaboAnalyst: a web server for metabolomic data analysis and interpretation. Nucleic Acids Res 37:W652–W660. doi:10.1093/nar/gkp356. 19429898 PMC2703878

[73] Stražar M, Temba GS, Vlamakis H, Kullaya VI, Lyamuya F, Mmbaga BT, Joosten LA, van der Ven AJ, Netea MG, de Mast Q. 2021. Gut microbiome-mediated metabolism effects on immunity in rural and urban African populations. Nat Comm 12:4845. doi:10.1038/s41467-021-25213-2. PMC8357928 34381036

[74] Xia J, Wishart DS. 2010. MSEA: a web-based tool to identify biologically meaningful patterns in quantitative metabolomic data. Nucleic Acids Res 38:W71–W77. doi:10.1093/nar/gkq329. 20457745 PMC2896187

