# Supplementary material for: Gut Microbial Succession Patterns and Metabolic Profiling during Pregnancy and Lactation in a Goat Model
Source: Microbiol Spectr. 2023 Jan 26;11(1):e02955-22. doi: 10.1128/spectrum.02955-22 (PMC9927511; doi:10.1128/spectrum.02955-22)
Supplement: Supplemental file 1 — Fig. S1 to S5 and Tables S1 and S4. Download spectrum.02955-22-s0001.pdf, PDF file, 1.7 MB [file spectrum.02955-22-s0001.pdf]

Supplementary Materials for

Gut microbial succession patterns and metabolic profile during pregnancy and lactation in goat model

Ke Zhang [a,1], Gongwei Liu [a,1], Yujiang Wu [b, c,1], Ting Zhang [a], Mengmeng Guo [d], Yu Lei [a], Xi Cao [a], Langda Suo [b, c], Daniel Brugger [e], Xiaolong Wang [a], Yuxin Yang [a*], Yulin Chen [a *]

[1]These authors contributed equally to this work.

*Correspondence authors.

Email addresses: Yulin Chen: chenyulin@nwafu.edu.cn (Y. Chen); yangyuxin2002@126.com (Y. Yang)

This PDF file includes:

Figures S1 to S5

Table S1 and Table S4

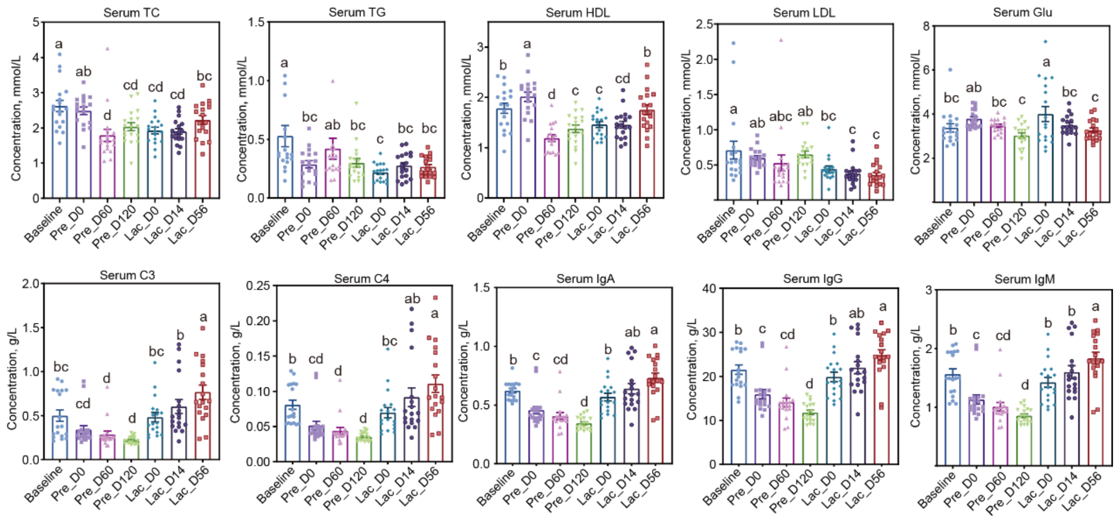

Fig. S1. The serum parameters during pregnancy and lactation in goats.

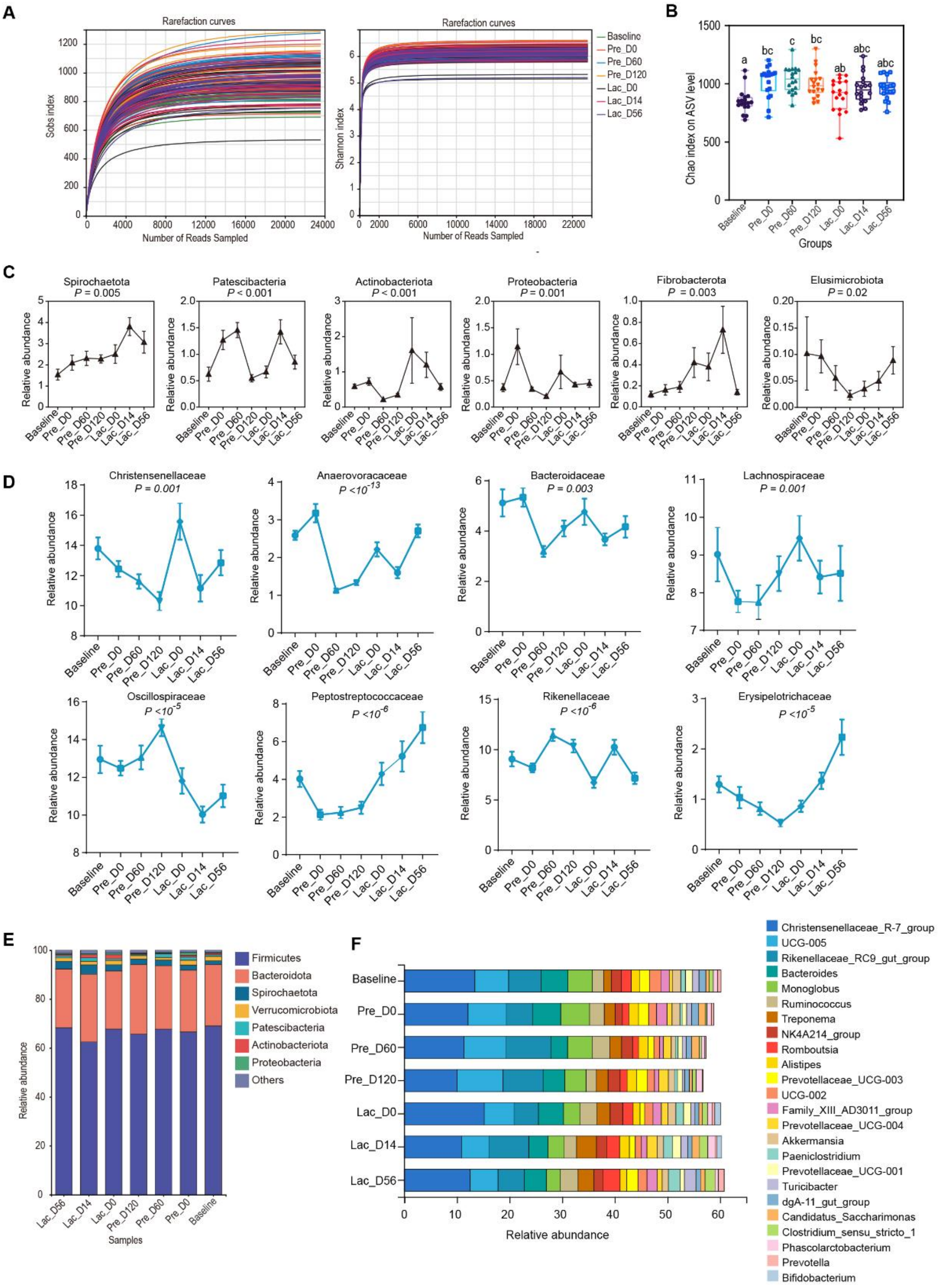

Fig. S2. The gut microbiota composition of the goat during pregnancy and lactation. (A) Rarefaction analysis of different samples based on Sob and Shannon index. (B) Chao index on ASV level of different group. (C-D) The relative abundance of core microbes in phyla and family level. The color-coded bar plots represent the average distribution of bacterial phyla (E) and genera (F), respectively. Only the dominant bacteria (with a relative abundance ≥1%) among gut bacteria are shown.

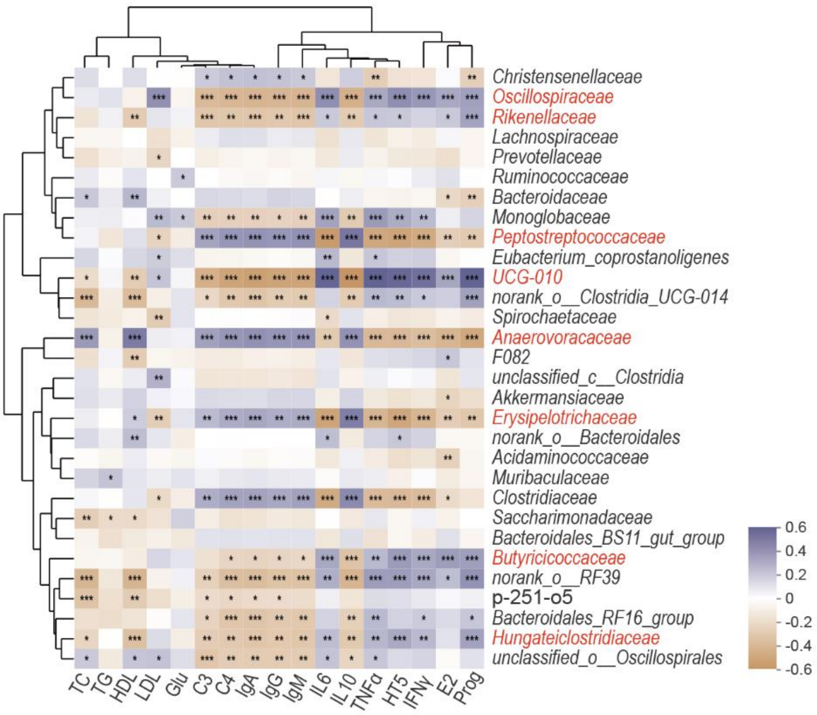

Fig. S3. Spearman correlation analysis between the different microbes in family level and serum index. * $P < 0.05$, ** $P < 0.01$, and *** $P < 0.001$.

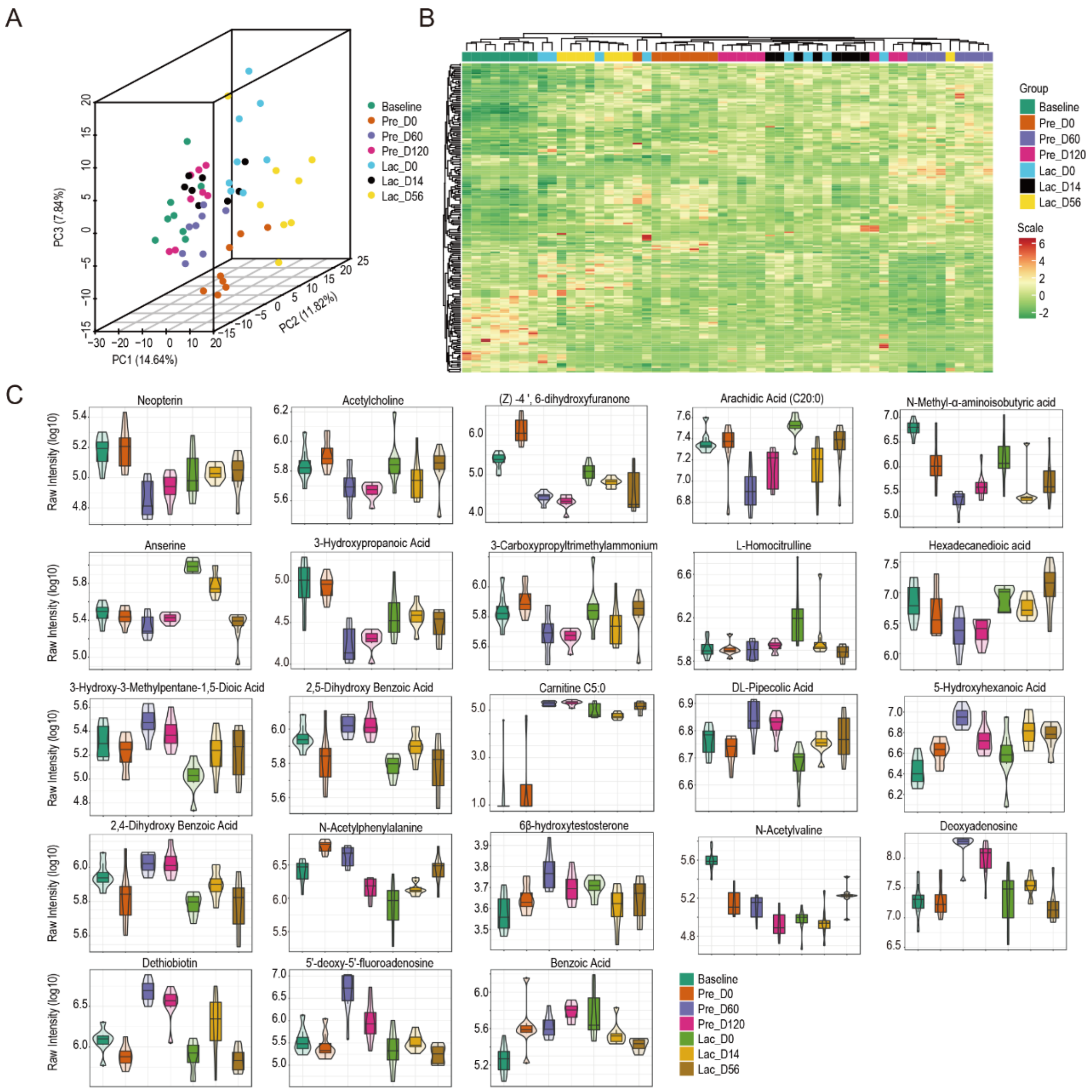

Fig. S4. Untargeted metabolomics cluster the goat faecal samples during the pregnancy and lactation. (A) The principal component analysis of all metabolic in seven groups. (B) The clustering heatmap of different metabolic in all samples. (C) The violin chart of differential metabolites in all time point.

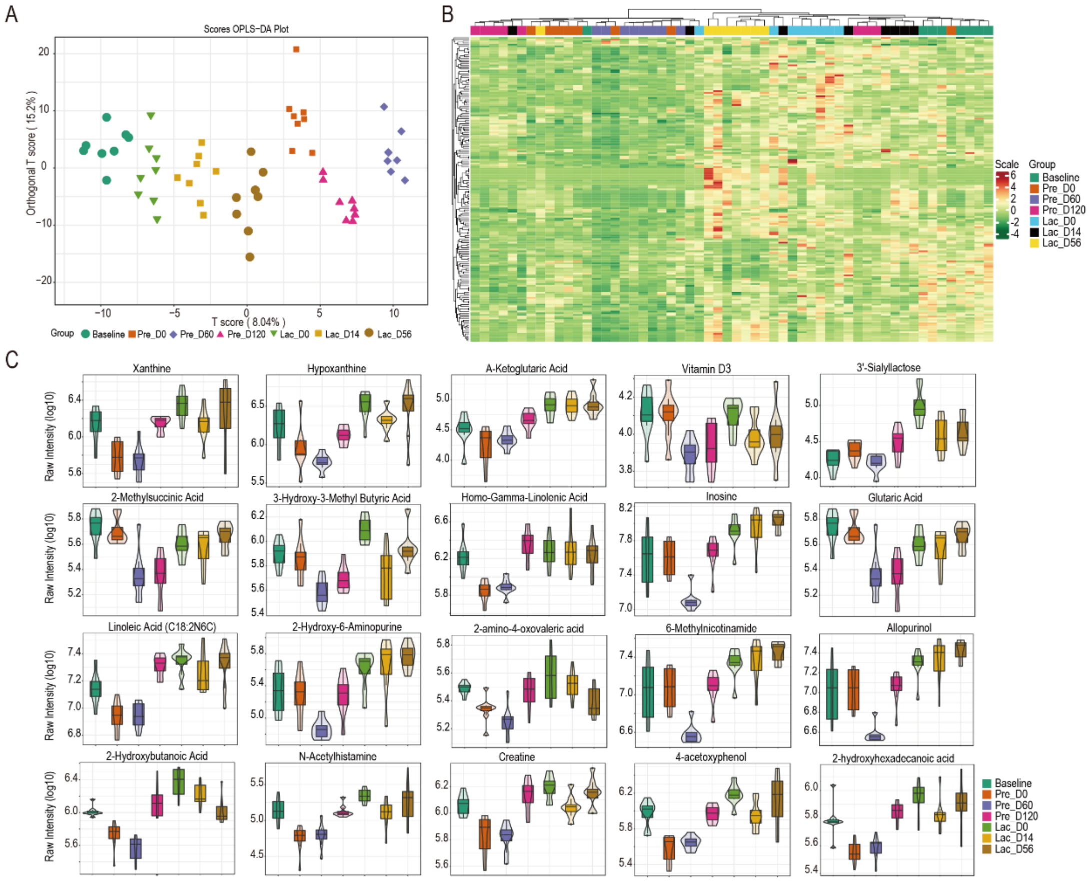

Fig. S5. Untargeted metabolomics cluster the goat serum samples during the pregnancy and lactation. (A) The OPLS-DA analysis distributed individual samples according to pregnancy and lactation stages. (B) The clustering heatmap of different metabolic in all samples. (C) The violin chart of differential metabolites in all time point.

Table S1. Ingredients of the experimental diets.

Item	Content (DM, %)
Ingredient	
Corn grain	47.08
Soybean meal	17.80
Wheat grain	8.00
Wheat Bran	5.00
Alfalfa	5.00
Highland barley	10.00
Methionine	0.02
Zeolite powder	0.50
Multivitamins	0.10
Yeast Extraction	3.00
Premix	1.00
Salt	1.00
Ca, P	1.50
Nutritional level (%)	
Dry matter	80.97
Crude protein	14.97
Crude fat	2.65
Crude fiber	7.21
Nitrogen free extract	51.91
Crude ash	2.73
Neutral detergent fiber	14.05
Acid detergent fiber	6.52

Table S4. The key metabolites of up-regulated at each sampling point.

Group	Formula	Compounds	Class I	CAS
1	C10H11NO3	N-Phenylacetylglycine	Amino acid and Its metabolomics	500-98-1
1	C13H16N2O4	Phenylacetyl-L-Glutamine	Amino acid and Its metabolomics	28047-15-6
1	C27H44O	Vitamin D3	CoEnzyme and vitamins	67-97-0
1	C5H8O4	2-Methylsuccinic Acid	Organic acid And Its derivatives	498-21-5
1	C5H8O4	Glutaric Acid	Organic acid And Its derivatives	110-94-1
1	C5H8O4	Ethylmalonate	Organic acid And Its derivatives	601-75-2
1	C7H13BaO10P	D-Sedoheptuiose 7-Phosphate	Carboxylic acids and derivatives	2646-35-7
1	C18H22O5S	estrone 3-sulfate	Hormones and hormone related compounds	481-97-0
1	C7H13NO3	N-Acetylvaline	Amino acid and Its metabolomics	3067-19-4
1	C6H13NO3S	Cyclamic acid	Organic acid And Its derivatives	100-88-9
1	C10H11NO3	N-(2-Methylbenzoyl) glycine	Amino acid and Its metabolomics	42013-20-7
1	C22H32O3	(±)4-HDHA	Lipids	90906-40-4
1	C20H32O3	(±)5-HETE	Lipids	-
1	C20H32O3	(±)9-HETE	Lipids	79495-85-5
1	C20H34O3	5-HETrE	Lipids	195061-94-0
1	C6H14O12P2	D-inositol-1,4-diphosphate	Alcohol and amines	74465-19-3
1	C7H13NO3	N-Isovaleroylglycine	Amino acid and Its metabolomics	16284-60-9
1	C13H16N2O4	N-γ-Acetyl-N-2-Formyl-5-Methoxykynurenamine	Amino acid and Its metabolomics	52450-38-1
1	C10H12N2O2	Cotinine N-Oxide	Heterocyclic compounds	36508-80-2
2	C6H13N3O3	L-Citrulline	Amino acid and Its metabolomics	372-75-8
2	C8H8O3	2-Methoxybenzoic Acid	Benzene and substituted derivatives	529-75-9
2	C24H40O4	Ursodeoxycholic Acid	Bile acids	128-13-2
2	C24H40O4	Hododeoxycholic acid	Bile acids	83-49-8

2	$C_{24}H_{40}O_4$	Chenodeoxycholic Acid	Bile acids	474-25-9
2	$C_7H_{11}N_3O_2$	1-Methylhistidine	Amino acid and Its metabolomics	332-80-9
2	$C_4H_4O_5$	Oxaloacetic acid	Organic acid And Its derivatives	328-42-7
2	$C_{23}H_{38}O_4$	23-deoxydeoxycholic acid	Bile acids	53608-86-9
2	$C_{24}H_{40}O_5$	Gamma-Mercholic Acid	Bile acids	547-75-1
2	$C_7H_4N_2O_7$	2-Hydroxy-3,5-dinitrobenzoic acid	Organic acid And Its derivatives	609-99-4
2	$C_{11}H_{11}NO_2$	3-Indolepropionic Acid	Heterocyclic compounds	830-96-6
2	$C_4H_9NO_3$	L-Homoserine	Amino acid and Its metabolomics	672-15-1
2	$C_9H_{10}O_2$	2'-Hydroxy-5'-methylacetophenone	Benzene and substituted derivatives	1450-72-2
2	$C_{30}H_{46}O_4$	18β-Glycyrrhetinic acid	Others	471-53-4
2	$C_{24}H_{40}O_5$	Beta-murine	Bile acids	2393-59-1
2	$C_{24}H_{40}O_3$	Lithocholic acid	Bile acids	434-13-9
2	$C_{18}H_{22}O_2$	Estrone	Hormones and hormone related compounds	53-16-7
2	C_9H_9N	3-methylindole	Heterocyclic compounds	83-34-1
2	$C_{20}H_{37}NO_4$	Carnitine C13:1	Lipids	-
2	$C_{18}H_{26}O_2$	Octapentaenoic acid	Lipids	-
3	$C_6H_{12}O_5$	L-Fucose	Carboxylic acids and derivatives	2438-80-4
3	$C_6H_{12}O_5$	L-Rhamnose	Carboxylic acids and derivatives	3615-41-6
3	$C_4H_8O_3$	3-Hydroxybutyrate	Organic acid And Its derivatives	300-85-6
3	$C_3H_4O_4$	Malonicacid	Organic acid And Its derivatives	141-82-2
3	$C_{18}H_{30}O_2$	A-Linolenic Acid(C18:3N3)	Lipids	463-40-1
3	$C_{16}H_{30}O_4$	Hexadecanedioic acid	Lipids	505-54-4
3	$C_{11}H_{14}N_2O_3$	Glycyl-phenylacrylic acid	Amino acid and Its metabolomics	721-66-4
3	$C_{18}H_{30}O_4$	9(S)-HpOTrE	Lipids	111004-08-1
3	$C_{18}H_{34}O_3$	Cis-9,10-epoxystearic acid	Lipids	2443-39-2
3	$C_{16}H_{32}O_3$	16-Hydroxyhexadecanoic acid	Lipids	506-13-8

3	C20H30O4	20-Carboxyarachidonic acid	Lipids	79551-84-1
3	C25H48NO7P	PysoPE 20:2	Lipids	-
3	C20H35NO3	Glycine linoleate	Lipids	-
3	C7H12N2O4	Nα-Acetyl-L-glutamine	Amino acid and Its metabolomics	2490-97-3
3	C11H14N2O3	Glycylphenylalanine	Amino acid and Its metabolomics	721-66-4
3	C19H38O4	1-Single Palm Essence	Lipids	542-44-9
3	C18H34O	9-octadecenal	Aldehyde, Ketones, Esters	5090-41-5
4	C7H15N3O3	L-Homocitrulline	Amino acid and Its metabolomics	1190-49-4
4	C6H14O6	Dulcitol	Carboxylic acids and derivatives	608-66-2
4	C10H14N2O5	Thymidine	Nucleotide and Its metabolomics	50-89-5
4	C11H11NO2	Methyl Indole-3-Acetate	Heterocyclic compounds	1912-33-0
4	C4H8O3	2-Hydroxybutanoic Acid	Organic acid And Its derivatives	600-15-7
4	C3H6O3	3-Hydroxypropanoic Acid	Organic acid And Its derivatives	503-66-2
4	C6H10O4	Adipic Acid	Organic acid And Its derivatives	124-04-9
4	C3H9N3O3S	Guanidinoethyl Sulfonate	Organic acid And Its derivatives	543-18-0
4	C23H39NO19	3'-Sialyllactose	Carboxylic acids and derivatives	35890-38-1
4	C12H15NO4	N-lactoyl-phenylalanine	Amino acid and Its metabolomics	-
4	C5H9NO3	N-Acetyl-L-alanine	Amino acid and Its metabolomics	97-69-8
4	C7H8O2	4-Hydroxybenzyl alcohol	Benzene and substituted derivatives	623-05-2
4	C8H8O3	Methylparaben	Benzene and substituted derivatives	99-76-3
4	C5H9NO3	N-acetyl-beta-alanine	Amino acid and Its metabolomics	3025-95-4
4	C8H8O3	2',4'-Dihydroxyacetophenone	Benzene and substituted derivatives	89-84-9
4	C5H9NO3	2-amino-4-oxovaleric acid	Organic acid And Its derivatives	4439-83-2
4	C5H6N2O2	Thymine	Nucleotide and Its metabolomics	65-71-4
4	C10H15NO2	2-(3,4-dimethoxyphenyl) ethanamine	Benzene and substituted derivatives	120-20-7
4	C8H8NO4P	Indole 3-phosphate	Heterocyclic compounds	13822-19-0

5	C9H10O3	Phenyllactate (Pla)	Organic acid And Its derivatives	828-01-3
5	C18H32O4	9-HpODE	Lipids	29774-12-7
5	C10H12N2O8	Orotidine	Nucleotide and Its metabolomics	314-50-1
5	C9H9NO4	Salicyluric acid	Benzene and substituted derivatives	487-54-7
5	C8H9NO2	Acetaminophen	Benzene and substituted derivatives	103-90-2
5	C5H9NO3	N-Propionylglycine	Amino acid and Its metabolomics	21709-90-0
5	C3H9NO	Trimethylamine N-Oxide	Alcohol and amines	1184-78-7
5	C9H19N3O3	L-Alanyl-L-Lysine	Amino acid and Its metabolomics	6366-77-4
5	C5H4N2O4	Orotic Acid	CoEnzyme and vitamins	65-86-1
5	C17H20N4O6	Riboflavin	CoEnzyme and vitamins	83-88-5
5	C5H11NO2	5-Aminovaleric Acid	Amino acid and Its metabolomics	660-88-8
5	C3H7N3O2	Guanidineacetic Acid	Organic acid And Its derivatives	352-97-6
5	C4H4O4	Maleic Acid	Organic acid And Its derivatives	110-16-7
5	C10H12N5O6P	Cyclic Amp	Nucleotide and Its metabolomics	60-92-4
5	C8H9NO	2-Phenylacetamide	Benzene and substituted derivatives	103-81-1
5	C3H9NO	1-Aminopropan-2-ol	Alcohol and amines	78-96-6
5	C14H19NO4	Carnitine ph-C1	Lipids	-
